# Optimal sizing and location of grid-interfaced PV, PHES, and ultra capacitor systems to replace LFO and HFO based power generations

**DOI:** 10.1038/s41598-024-57231-7

**Published:** 2024-04-13

**Authors:** Isaac Amoussou, Emmanuel Tanyi, TakeleFerede Agajie, Baseem Khan, Mohit Bajaj

**Affiliations:** 1 Department of Electrical and Electronic Engineering, Faculty of Engineering and Technology, University of Buea, P.O. Box. 63, Buea, Cameroon; 2 Department of Electrical and Computer Engineering, Debre Markos University, P.O. Box 269, Debre Markos, Ethiopia; 3 Department of Electrical and Computer Engineering, Hawassa University, P.O. Box 05, Hawassa, Ethiopia; 4 Department of Electrical Engineering, Graphic Era (Deemed to Be University), Dehradun, 248002 India; 5 Hourani Center for Applied Scientific Research, Al-Ahliyya Amman University, Amman, Jordan; 6 Graphic Era Hill University, Dehradun, 248002 India; 7 Applied Science Research Center, Applied Science Private University, Amman, 11937 Jordan

**Keywords:** LFO, HFO, Thermal power plant, LOLP, LPSP, TAC, Metaheuristics, Engineering, Electrical and electronic engineering, Energy infrastructure

## Abstract

The impacts of climate change, combined with the depletion of fossil fuel reserves, are forcing human civilizations to reconsider the design of electricity generation systems to gradually and extensively incorporate renewable energies. This study aims to investigate the technical and economic aspects of replacing all heavy fuel oil (HFO) and light fuel oil (LFO) thermal power plants connected to the electricity grid in southern Cameroon. The proposed renewable energy system consists of a solar photovoltaic (PV) field, a pumped hydroelectric energy storage (PHES) system, and an ultra-capacitor energy storage system. The economic and technical performance of the new renewable energy system was assessed using metrics such as total annualized project cost (TAC), loss of load probability (LOLP), and loss of power supply probability (LPSP). The Multi-Objective Bonobo Optimizer (MOBO) was used to both size the components of the new renewable energy system and choose the best location for the solar PV array. The results achieved using MOBO were superior to those obtained from other known optimization techniques. Using metaheuristics for renewable energy system sizing necessitated the creation of mathematical models of renewable energy system components and techno-economic decision criteria under MATLAB software. Based on the results for the deficit rate (LPSP) of zero, the installation of the photovoltaic field in Bafoussam had the lowest TAC of around 52.78 × 10^6^€ when compared to the results for Yaoundé, Bamenda, Douala, and Limbe. Finally, the project profitability analysis determined that the project is financially viable when the energy produced by the renewable energy systems is sold at an average price of 0.12 €/kWh.

## Introduction

Electricity is always generated by converting non-renewable energy sources, such as heavy fuel oil, light fuel oil, coal, and natural gas, on the one hand, and renewable energy sources, such as solar radiation, on the other. Fossil fuels make up a significant proportion of the energy sources used in power generation worldwide. The consequences of using such polluting energy sources to generate electricity are both economic and environmental. Indeed, the energy sector is the largest emitter of greenhouse gases into the atmosphere. In 2020, this sector alone generated 20 GtCO_2_, accounting for approximately 37% of total greenhouse gas emissions into the atmosphere^1^. Furthermore, in 2021, the electricity and heat production sectors accounted for 46% of the increase in greenhouse gas emissions^2^. The release of greenhouse gases into the atmosphere accelerates global warming, with tragic consequences for human societies. If nothing is done, extreme climatic phenomena such as storms and cyclones will become recurrent, as will droughts on every continent, including Africa. The competitiveness of diesel, HFO, and natural gas-fired power plants is greatly affected by global hydrocarbon prices. These prices have a general upward trend and fluctuate according to factors exogenous to the hydrocarbon extraction sector, such as wars and health crises. Finally, the depletion of hydrocarbon reserves, predicted by some studies to occur in a few decades^3^, is forcing human societies to think about alternatives to these non-renewable energy sources. Electricity generation system architectures must, therefore, progressively integrate renewable energy production systems in order to replace thermal power plants with renewable energy plants.

Renewable energy sources can be categorized into controllable sources such as hydroelectricity, biomass, and geothermal energy and intermittent sources such as solar and wind power. The total installed capacity of solar energy systems has grown steadily, as has the maturity of the technologies used in renewable energy systems. The technologies used in solar systems are increasingly mature and profitable. In fact, according to the International Renewable Energy Agency (IRENA), the cost of solar photovoltaic (PV) modules has fallen by around 93% over the last decade, and the levelized cost of energy (LCOE) has fallen by 85% for grid-connected applications over the same period^4^. This has encouraged the installation of photovoltaic systems for a variety of applications. As a result, the average growth rate for photovoltaic systems rose to 34% between 2010 and 2020^5^. The worldwide installed capacity of solar PV systems is estimated to be around 939 GW in 2021^6^.

Annual electricity production in Cameroon is primarily derived from hydroelectric and thermal power plants. Hydroelectric power accounted for 70% of total consumption in Cameroon in 2021^7^. Thermal power plants supply the remaining 30%. Given the negative environmental impact of fossil fuels and their increasing scarcity, the Cameroonian government needs to implement a policy of replacing existing thermal power plants with renewable energy systems, in particular solar photovoltaic systems. Cameroon receives abundant sunshine across its entire territory. Indeed, the average irradiance across the country ranges from 5.8 kWh/m^2^ in the north to 4.9 kWh/m^2^ in the south^8^.

However, switching from thermal to renewable energy plants presents a number of technical and economic challenges. Indeed, replacing thermal power plants, whose energy production is perfectly controllable, with solar photovoltaic systems, which are inherently intermittent, may lead to production-demand mismatches. The uncontrollable and unpredictable nature of solar radiation can cause stability issues in Cameroon's aging power grid. Energy storage systems can be combined with renewable energy systems to increase their reliability^9^. Storage system implementation bridges the gap between production and consumption by delivering the energy required to meet demand in the event that solar power systems fail. Lithium batteries^10–12^, hydrogen^13–15^, compressed air energy storage (CAES)^16–18^, and pumped hydro energy storage (PHES)^19–21^ are examples of storage systems that can be integrated with PV power plants to balance the supply and demand of energy. PHES is the most widely used storage system in the world, particularly for large-scale applications^22,23^. This storage technology has a relatively long lifespan and low energy production costs compared to alternative storage technologies. The presence of favorable topographical features for the installation of PHES systems^24^ and the accessibility of water resources^25^ are essential prerequisites. Nevertheless, PHES systems require a few minutes of transient time before they can meet load demand^26^. Storage systems with high power density^27^ and millisecond response times, such as ultra-capacitors (super-capacitors), are required to ensure grid reliability. However, integrating storage systems into PV power plants raises the capital and operating costs of renewable energy installations. Solar PV and energy storage systems must be optimally dimensioned in order to be competitive and attractive. To size renewable energy systems optimally, several methods are used. Two sizing methods were discovered to be the most widely used in the literature reviewed. These are HOMER^28,29^, and meta-heuristics^30,31^. Several comparative studies of the results provided by the two sizing methods show that meta heuristics provide the best results in terms of system cost and reliability when compared to HOMER software^32–34^.

Several studies have been conducted to investigate the optimal sizing of renewable energy systems using meta-heuristic algorithms based on economic and technical criteria. Thus, in Ref.^35^, three algorithms, namely the whale optimization algorithm (WOA), particle swarm optimization (PSO), and fire fly (FF), were used to minimize the cost of energy (COE) and the loss of power supply probability (LPSP) for a hybrid wind/PV/biomass/PHES system isolated from the electrical grid. The WOA produced the best results in this study. In Ref.^36^, the effectiveness of a modified crow search algorithm (CSA) in reducing fuel consumption in an autonomous PV/diesel/PHES system was investigated. The modified CSA results were compared to the results obtained using the genetic algorithm (GA) and PSO. It has been discovered that the results obtained using modified CSA outperform those obtained using GA and PSO. The optimal sizing of a hybrid solar PV, biogas, and PHES system isolated from the electrical grid using metaheuristics such as the water cycle algorithm (WCA), moth flame optimization (MFO), and GA using total net present cost (TNPC) and loss of load probability (LLP) criteria has been completed in Ref.^37^. The WCA algorithm outperformed the others. WOA, WCA, grey wolf optimizer (GWO), and salp swarm algorithm (SSA) were used in Ref.^38^ to optimize the sizing of a grid-connected PV-Wind-PHES hybrid system with the goal of minimizing the COE. Under a well-defined loss of power supply probability, the WOA algorithm provided the best energy cost. The MOGWO algorithm was used in^39^ to optimize the sizing of a hybrid storage system comprised of PHES (long-term storage) and battery (short-term storage) integrated with PV and wind renewable energy systems. This study discovered that the hybrid storage system outperformed PHES and batteries alone in terms of energy cost. In Ref.^40^, the Multi-Objective Particle Swarm Optimization (MOPSO) algorithm has been used to optimize the sizing of a PV-Wind-PHES hybrid system with the goal of minimizing the LCOE and LPSP. Furthermore, the authors determined the optimal sizing for hybrid solar PV-wind renewable energy systems with PHES while taking LCOE into account^41^. According to the findings, a hybrid PV-wind system with PHES has the highest system capacity and the lowest LPSP and COE^42^. It is possible to compensate for the intermittent nature of renewable energy sources by using PHES technology to support a microgrid hybrid solar-wind system^43^. Several studies have examined the integration of fast-response storage systems, such as superconducting magnetic energy storage (SMES), with the PHES^44–46^. The purpose of the SMES system was to provide energy as needed during the transitional periods necessary for the start-up of the primary storage system, the PHES. The integration of supercapacitors with batteries in Ref.^47^ resulted in the development of a dependable renewable energy system that can efficiently respond to demand. Fast-response storage systems are essential for the widespread integration of renewable energy systems into the energy mix of nations worldwide. Finally, as intermittent renewable energies are integrated into the power grid, energy management becomes more complex. Energy management strategies for microgrids, including electric car energy storage as virtual system storage, were proposed in Refs.^48–50^, with the primary goals of reducing grid dependence and lowering energy costs. This work additionally seeks to create an energy management strategy for Cameroon's southern interconnected grid, allowing for large-scale integration of PV systems and PHES power plants.

The main goal of this work is to propose a new architecture for the electricity production system, which would replace the HFO and LFO power plants linked to the SIG with renewable energy systems comprised of a PV field, a PHES power plant, and an ultra-capacitor battery system. LOLP, LPSP, and TAC were used as technical–economic criteria to assess the reliability and investment required for the installation of new renewable energy systems. MOBO, MSSA, MOALO, MOPSO, SPEA2, and MOAVOA optimization techniques were used to achieve optimal and economically appealing results. The primary contributions of this work in comparison to previous studies can be succinctly summarized as follows:

1. Mathematical modelling of solar PV, PHES, and ultra-capacitor systems and their optimal dimensioning using multi-objective optimization algorithms as potential replacements for HFO and LFO thermal power plants connected to Cameroon's southern interconnected grid were conducted. On this basis, the ultra-capacitors were specifically sized to serve as the fast-response energy storage system required during the transition period necessary for the start-up of the PHES system.
2. The impact of the geographical location of the solar PV field on the total annualized cost was investigated in order to inform decision-makers about the best areas for PV installation in Cameroon's southern region.
3. The payback period has been used to determine an energy selling price, which ensures the profitability of the proposed new renewable energy system.
4. This study can serve as a solid scientific foundation for the development and implementation of large-scale solar PV and PHES systems in Cameroon.

The subsequent sections of the paper are structured in the following manner: Section "Overview of the study area" provides an overview of the geographical area being studied. Section "Proposed renewable energy system" includes a schematic and description of the proposed renewable energy system, along with mathematical modeling of its components. Section "Evaluation parameters" focuses on modeling the evaluation parameters, including technical and financial aspects. Section "Formulation of problems and Optimization" presents the formulation of the optimization problem. Section "Optimization Algorithms" describes the metaheuristic optimization techniques used in this study. Section "Proposed system operational strategies" discusses the energy management of the renewable system. Section "Result and discussion" presents the results and includes a discussion. Finally, Section "Conclusion and future research directions" provides a conclusion, followed by the references.

## Overview of the study area

### Southern interconnected grid

**Figure 1:**
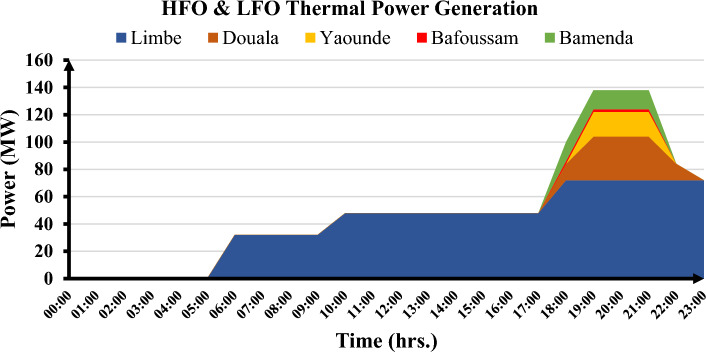
SIG thermal power plant production over a one-day period.

**Figure 2:**
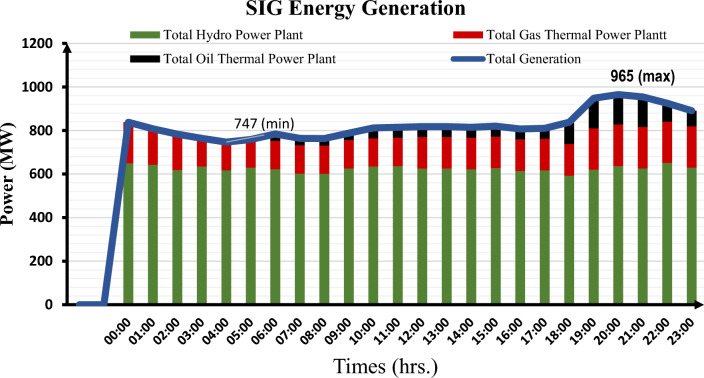
Total energy production on SIG over a one-day period.

The Southern Interconnected Grid (SIG) of Cameroon is the most extensive, serving approximately six of the country's ten regions. These are the Littoral, Center, South, West, North-West, and South-West regions. The SIG is made up of transmission lines rated at 225 kV/90 kV, substations rated at 225 kV/90 kV with 225 kV/90 kV transformers, distribution substations rated at 90 kV/30 kV and 90 kV/15 kV, and low-voltage distribution lines. The Edea and Songloulou hydroelectric dams, as well as the Mem'vele hydroelectric dam, account for a significant portion of the total capacity of power generation units connected to this grid. The thermal power plants were built primarily to meet rapidly increasing demand on the one hand and to provide backup power on the electricity grid during peak periods on the other. Thermal power plants linked to the SIG are managed by either the parastatal ENEO or independent producers. In contrast to the Lagdo hydroelectric dam in Cameroon's semi-arid north, hydroelectric dams in the south, particularly in the Sanaga basin, are less affected by global warming. There are numerous thermal power plants connected to the southern interconnected grids that run on three types of fuel: gas, HFO, and diesel. Given the low cost of gas-fired power generation, the 216 MW gas-fired power station is the most commonly used, producing power at or above 75 MW continuously. Other thermal power plants assist in power grid stabilization during peak demand in specific areas or urban centers. It should also be noted that, due to the extremely high cost of diesel power generation, HFO power plants are in higher demand than LFO thermal power plants. Diesel-fired power plants are used as a last resort, particularly during peak periods on the electricity grid between 6 p.m. and 10 p.m. In 2021, ENEO's thermal power plants generated approximately 7% of the total energy generated by all generation systems^7^. Figures 1 and 2 depict the energy production profiles of the different thermal power plants, as well as the energy flow on the southern power grid.

### Solar resource assessment

The majority of Cameroon's territory receives abundant sunlight, making it an ideal location for the installation of small and large-scale solar photovoltaic systems. The average annual solar irradiation ranges from 4.9 to 5.8 kWh/m^2^/day in the south and north, respectively^8^. As reviewed in different research works, the northern regions of the country have significant potential for the installation of large-scale solar PV fields. Other regions of Cameroon also have significant potential for solar photovoltaic resources. Indeed, the western and north-western regions, along with the rest of the country, have significant solar PV resource potential. The relatively mild average temperatures in some regions make solar PV systems ideal for installation. According to this study^51^, the cities of Bafoussam and Bamenda have a significant capacity for solar photovoltaic energy generation. Existing initiatives aim to exploit this untapped potential. The Cameroonian government and international partners are examining projects to install solar PV power plants in both the country's north and south^8^.

**Figure 3:**
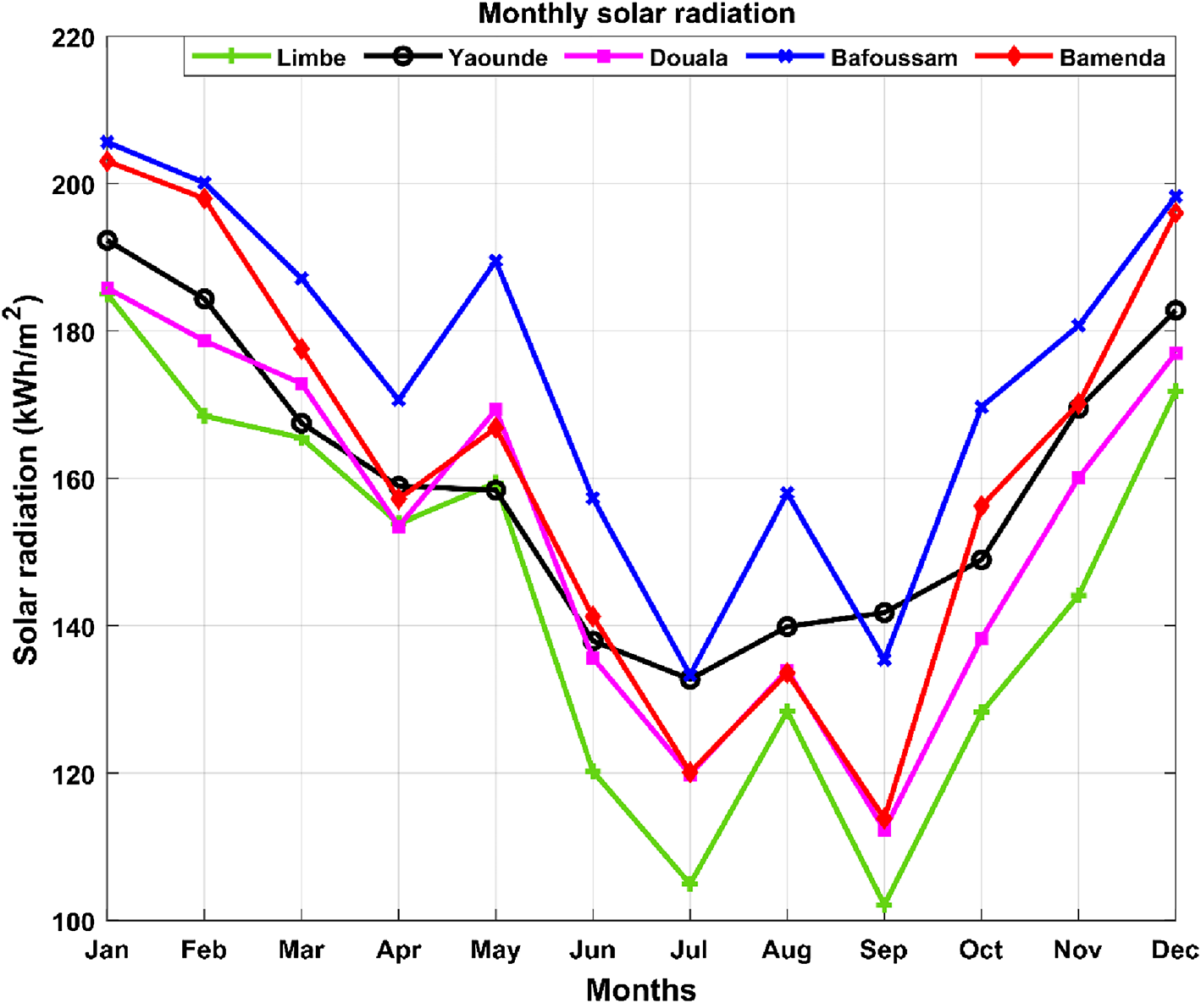
Monthly solar radiation for the selected areas.

Figure 3 depicts monthly solar radiation data for localities with thermal power plants in southern Cameroon. The data in question were derived from^52^. This figure reveals that solar PV has a strong potential in all geographical zones of Cameroon.

Figure 3 shows the minimum monthly solar radiation for Limbe, Douala, Bamenda, Yaoundé, and Bafoussam as 102.10 kWh/m^2^, 112.29 kWh/m^2^, 113.83 kWh/m^2^, 132.74 kWh/m^2^, and 133.32 kWh/m^2^, respectively. Limbe, Douala, Bamenda, Yaoundé, and Bafoussam have maximum monthly solar radiation measurements of 184.98 kWh/m^2^, 185.81 kWh/m^2^, 203.03 kWh/m^2^, 192.30 kWh/m^2^, and 205.62 kWh/m^2^, respectively. As a result, Bafoussam has the best insolation conditions for solar system installation when compared to Bamenda, Yaoundé, Douala, and Limbe.

### Assessment of suitable locations for PHES systems in Cameroon

The availability of water resources and space at a sufficiently high altitude to provide sufficient potential energy determines the potential for large-scale installation of PHES systems. According to a study^53^, Cameroon has significant potential for PHES system installation, particularly in the northern, western, and north-western regions. The total capacity of PHES systems that could be installed, according to the same study, is nearly 33.36 GWh. There are dozens of sites in the country's south with favorable characteristics for the installation of PHES systems. Among the dozens of sites suitable for PHES systems, the following are the most important in terms of storage capacity: The Enep site has a capacity of 4.998 GWh and a height of 560 m, the Bamendjin site has a capacity of 4.804 GWh and a height of 549 m, and the Banakuma site has a capacity of 4.861 GWh and a height of 505 m^53,54^. A ranking of the various sites potentially suitable for installation based on multiple criteria revealed that the Enep site came in first place.

## Proposed renewable energy system

**Figure 4:**
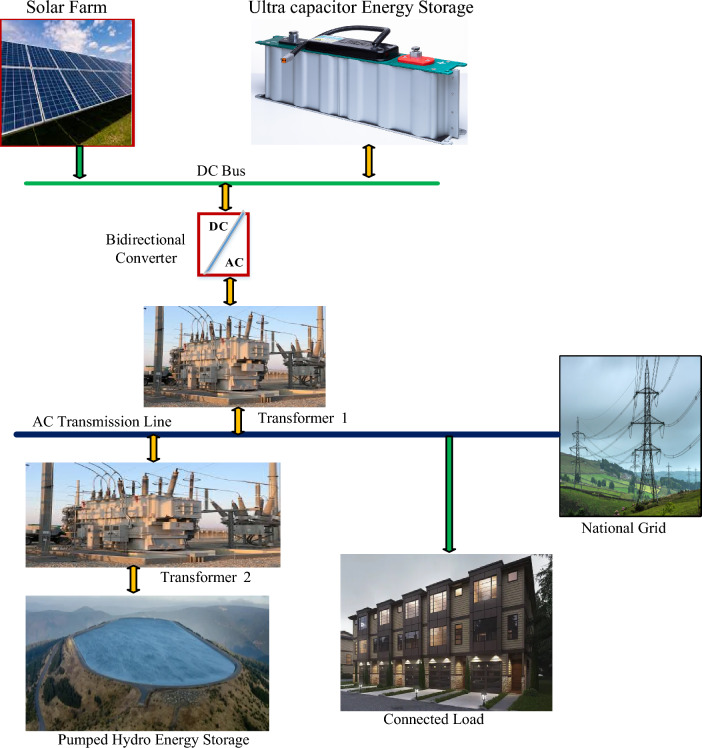
Proposed renewable energy systems interfaced to the grid with PHES and ultra-capacitors storage systems.

The proposed renewable energy system consists of a PV field, a PHES power plant, and an ultra-capacitor battery system functioning as a fast-response storage system. The layout proposed for the integration of renewable energy resources with the PHES and ultra-capacity energy storage system is illustrated in Fig. 4.

### Mathematical modeling of RES

A comprehensive understanding of the mathematical and economic models pertaining to renewable energy production sources and storage systems is imperative for the efficient sizing of each constituent component.

#### Solar photovoltaic generation system

In the literature, several mathematical models are used to calculate the power supplied by photovoltaic solar panels. The mathematical model utilized in this work has been used in several previous studies, including^55,56^. The model incorporates hourly irradiation and temperature data, as well as the surface area and efficiency of solar PV panels. It is determined by Eq. (1)^57,58^.

1 $${P}_{PV}(t)={N}_{PV}\times A\times G(t)\times {\eta }_{pv}$$

Here, $A$ represents the surface area of the solar panel,${N}_{PV}$ represents the number of solar panels,$G(t)$ represents the hourly irradiance of the site chosen for solar array installation, and ${\eta }_{pv}$ represents the efficiency of the solar panel. Equation (2) expresses this efficiency^55,57^:

2 $${\eta }_{pv}={\eta }_{r}\times {\eta }_{pc}\times \left[1-\beta \left({T}_{a}+\left(\frac{NOCT-20}{800}\right)\times G(t)-{T}_{cref}\right)\right]$$

where $\beta$ is the temperature constant relative to the photovoltaic cells and ${\eta }_{r}$ is the photovoltaic module's reference efficiency. It is defined as the ratio of the module's maximum power produced to the solar radiation power collected.

${\eta }_{pc}$ is the degradation factor that takes into account the optimal operating point of the modules when using an MPPT converter. ${T}_{cref}$ is the reference temperature of the photovoltaic cells in the solar panel, ${T}_{a}$ is the ambient temperature at the site, and NOCT is a solar panel-specific characteristic.

#### Energy storage system

The storage system consists of ultra-capacitors and a PHES power station. During the PHES system's transitory switch-on phase, the ultra-capacitors are solely needed for fast response to demand. The PHES system was designed for long-term storage.

##### Power balance

The Power balance here is the difference between the energy production from the solar ${P}_{PV}$ field and the energy demand met by the thermal plants ${P}_{DT}$.

3 $${E}_{B}\left(t\right)={P}_{PV}\left(t\right)\times {\eta }_{inv}-{P}_{DT}(t)$$

##### Ultra-capacitor

When the PV field output is insufficient to fulfill demand and the PHES switch-on time, the ultra-capacitor storage system steps in. The transitory time of the PHES system switch-on is estimated to be three minutes^59^ in this work. Because the simulation step is an hour, it is anticipated that the ultra-capacitors will only deliver the energy equivalent to the three-minute transient period of the PHES system when it is in demand. The ultra-capacitors are charged when the energy produced by the PV system exceeds demand. Equation (4) describes the energy flow in ultra-capacitors and is inspired by that proposed in Ref.^47^.

4 $${E}_{UC}\left(t\right)=\left(1-\delta \right)\times {E}_{UC}\left(t-1\right)+{\eta }_{UC}\times {\Delta tP}_{UC}(t)$$

This energy flow in the ultra-capacitor storage system is subject to the constraints described below:

5 $$\left\{\begin{array}{l}{E}_{UC}^{min}\le {E}_{UC}\left(t\right)\le {E}_{UC}^{max}\\ 0\le \left|{P}_{UC}(t)\right|\le {P}_{UC}^{max}\end{array}\right.$$

Here, ${E}_{UC}\left(t\right)$ represents the energy present in the ultracapacitors at time t,$\delta$ is the self-discharge rate of ultra-capacitors,${E}_{UC}\left(t-1\right)$ represents the energy available at time t-1, ${E}_{UC}^{min}$ represents the minimum energy imposed, ${E}_{UC}^{max}$ represents the rated capacity of the ultracapacitor system, ${P}_{UC}(t)$ represents the power supplied/charged and ${P}_{UC}^{max}$ represents the maximum rated power of the ultra-capacitors.When charging, ${P}_{UC}(t)$ is positive, and when discharging, ${P}_{UC}(t)$ is negative.

##### Pumped hydro energy storage system

The PHES plant operates in two modes: water pumping when the energy balance is more significant than zero and energy generation when the energy balance is less than zero.

(a) PHES generating mode: If ${E}_{B}(t)<0$, the demand exceeds the output of the PV and wind systems. In this instance, the energy must come from the PHES system. Equations (6) and (7) are derived from the models used in the papers^38,56^ and represent the operation of the PHES in generating mode.

6 $${E}_{PHES}^{dis}\left(t\right)=\mathit{min}\left\{\left(\frac{V(t-1)-{V}_{min}}{3600}\right)\times g\times \rho \times {\eta }_{t}\times H,min\left({P}_{PHSn},\left|{E}_{B}(t)\right|\right)\right\}$$

7 $${q}_{dis}(t)=\frac{{E}_{PHES}^{dis}(t)}{g\times \rho \times {\eta }_{t}\times H}$$

Here, it is assumed that the water density $\rho$ and gravitational constant $g$ are 1000 m^3^/kg and 9.81 m/s^2^, respectively. $H$ equals the sum of the water level height in the upper reservoir and the elevation of the upper reservoir's location above the lower reservoir. It is determined using Eq. (8). The height caused by the water level in the upper tank is dependent on the structure used to build it. Equation (9) permits its evaluation for a particular type of construction structure. This additional height is disregarded in this study due to the height h of the chosen site in relation to the lower reservoir, which is several hundred meters.

8 $$hadd(t)=\frac{V(t-1)}{area}$$

9 $$H(t)=h+hadd(t)$$

Equation (10) determines the power ${P}_{PHES}$ supplied by the PHES system to the loads^60^.

10 $${P}_{PHES}(t)={q}_{dis}\times g\times \rho \times {\eta }_{t}\times H$$

(b) PHES Pumping Mode: In the event that ${E}_{B}(t)>0$, it indicates the presence of surplus energy within the power grid. In the event that the upper reservoir is not at maximum capacity, the PHES system transitions into a pumping mode until the upper reservoir reaches its full capacity. Equations (11) and (12) are derived from the models used in the papers^38,56^ and represent the operation of the PHES in pumping mode.

11 $${E}_{PHES}^{ch}(t)=\mathit{min}\left\{\left(\frac{{V}_{max}-V(t-1)}{3600}\right)\times g\times \rho \times H\times \frac{1}{{\eta }_{P}}, {\text{min}}\left({P}_{PHSn},{E}_{B}(t)\right)\right\}$$

12 $${q}_{ch}(t)=\frac{{\eta }_{P}\times {E}_{PHS}^{ch}}{g\times \rho \times H}$$

Here, ${\eta }_{P}$ refers to the efficiency of the PHES system in pumping mode.

In pumping mode, the power consumed over time by the PHES system is calculated using Eq. (13)^60^.

13 $${P}_{Pump}(t)=\frac{\rho \times g\times {q}_{ch}(t)\times H}{{\eta }_{P}}$$

##### Model of the reservoirs

Equation (14) estimates the quantity of water present in the upper reservoir.

14 $$V(t)= \text{ } (\text{1} - \gamma )\times V(\text{t-1}) + \, \Delta {\text{t}}\times ({q}_{\text{ch}}(t)-{q}_{dis}(t))$$

Here, $\gamma$ represents the rate of evaporation and leakage over time.

The water volume in the upper reservoir fluctuates within the predetermined minimum and maximum limits.${V}_{min}\le V(t)\le {V}_{max}$

Equation (15) is used to compute the total energy stored in the upper reservoir, ${E}_{C}$, expressed in kWh^61,62^:

15 $${E}_{C}=\frac{{\eta }_{t}\times g\times \rho \times {V}_{max}\times H}{3.6\times 1{0}^{6}}$$

##### Surplus energy

Excess energy exists only when ${E}_{B}\left(t\right)>0$ and may be estimated using Eq. (16).

16 $${E}_{S}\left(t\right)={E}_{B}\left(t\right)-{E}_{PHS}^{ch}\left(t\right)-{\Delta tP}_{UC}(t)$$

##### Inverter

The inverters must be capable of converting the DC power output of the PV array and ultra-capacitors to AC.

17 $$\left\{\begin{array}{l}{P}_{inv}\left(t\right)\ge {P}_{PV}\left(t\right)+{P}_{UC}\left(t\right)\\ {P}_{invn}=SF\times {\text{max}}\left({P}_{PV}\left(t\right)+{P}_{UC}\left(t\right)\right)\end{array}\right.$$

Here, $SF$ represents the safety factor and is greater than 1, ${P}_{invn}$ represents the nominal capacity of the inverters*.*

##### Power flow on the grid

Equation 18 expresses the maximum power transmitted through the electrical grid. Hydroelectric power stations serve as the primary source of electricity for the southern interconnected grid.

18 $${P}_{grid}\left(t\right)={P}_{Hydro}\left(t\right)+{P}_{GasT}\left(t\right)+{P}_{PV}(t)$$

In this equation, ${P}_{Hydro}$ represents hydroelectric power, while ${P}_{GasT}$ represents natural gas-fired power plants. The amount of power flowing through the grid must not exceed the grid's maximum capacity (${P}_{grid}^{max}$).

19 $${P}_{grid}\left(t\right)\le {P}_{grid}^{max}$$

If the maximum grid capacity is exceeded, the output of the photovoltaic system is curtailed.

## Evaluation parameters

Sizing renewable energy systems requires decision criteria. These parameters are classified into two types: economic and financial decision criteria and technical criteria for determining system reliability.

### Reliability of the system

In this study, two reliability criteria were considered. These are loss of power supply probability (LPSP) and loss of load probability (LOLP).

#### Loss of power supply probability (LPSP)

The loss of power supply probability is a criterion that estimates the rate of energy deficit relative to demand over a given time period. This reliability criterion has been used in a number of studies^63–66^. It is calculated using Eq. (20).

20 $$LPSP=\frac{{\sum }_{i:1}^{8784}{P}_{DT}\left(t\right)-{{\eta }_{inv}\times P}_{PV}\left(t\right)-{P}_{PHES}\left(t\right)-{\Delta tP}_{UC}(t)}{\sum_{1:1}^{8784}{P}_{DT}}$$

#### Loss of Load Probability (LOLP)

A criterion assessing the hourly deficit rate has been established to assess the reliability of new renewable energy systems. The LOLP criteria is determined by Eqs. (19) and (20)^67,68^.

21 $$LOLP=y\left\{f(x)\le 0\right\}=\frac{1}{N}\sum_{1}^{t}{F}_{y}$$

22 $$f\left(x\right)={P}_{PV}\left(t\right)+{P}_{PHES}\left(t\right)+{\Delta tP}_{UC}(t)-{P}_{DT}(t)$$

In this instance, N = 8784 is the simulation period, and F is the failure frequency of the response y when it is negative.

### Economic model

Several economic criteria were taken into account in this work. These are the total annualized cost (TAC) of the project over its lifetime, the levelized cost of energy (LCOE) assessed for each energy production system, and the net present value (NPV) assessed for each configuration studied.

#### Total annualized cost (TAC)

The TAC comprises the cumulative sum of the annual capital cost (${C}_{CAP}$), the yearly maintenance and operating costs (${C}_{O\&M}$), and the annual replacement cost (${C}_{REP}$)^69,70^.

23 $$TAC={C}_{CAP}+{C}_{REP}+{C}_{O\&M}$$

The total annualized cost over the study period of the PV system and energy storage was evaluated.

(a) PV system: The total annualized cost of the PV system includes the initial cost and the cost of operations and maintenance. Considering the duration of the project (25 years) considered in this study, no component replacement will be made. It is calculated by Eq. (24):

24 $${TAC}_{PV}={N}_{PV}\times {C}_{cap}^{PV}\times CRF+{N}_{PV}\times {C}_{O\&M}^{PV}$$

where ${N}_{PV}$ represents the number of solar panels, ${C}_{cap}^{PV}$ represents the initial cost of the solar panels, ${C}_{O\&M}^{PV}$ represents the cost of operations and maintenance of the PV systems.Thecost of capital recovery factor $CRF$ depends on the rate of real interest rate and the project lifetime. It is provided by Eq. (25)^69,70^.

25 $$CRF=\frac{r{\left(1+r\right)}^{N}}{(1+r{)}^{N}-1}$$

Here $r$ represents the real interest rate, $N$ represents the considered study duration. The real interest rate depends on the nominal interest rate ${i}_{n}$ and the inflation rate ${i}_{f}$^69,71^.

26 $$r=\frac{{i}_{n}-{i}_{f}}{1+{i}_{f}}$$

(b) Inverters: The total life cycle cost of the inverters includes the capital cost, replacement cost, maintenance and operation costs, and salvage cost. The lifespan of the inverters considered here is 15 years. They are, therefore, replaced only once during the lifetime of the project.

27 $${TAC}_{inv}={{P}_{invn}\times (C}_{cap}^{inv}\times CRF+{C}_{rep}^{inv}\times {k}_{r}\times CRF+{C}_{O\&M}^{inv})$$

where ${P}_{invn}$ represents the nominal power of the inverter, ${C}_{cap}^{inv}$ represents the initial cost of the inverter, ${C}_{O\&M}^{inv}$ represents the cost of operations and maintenance of the inverters, ${C}_{rep}^{inv}$ represents the replacement cost of the inverter, and ${k}_{r}$ represents the replacement cost discount factor. It depends on the real interest rate $r$ and the component's life span $n$. It is calculable using Eq. (28)^72^.

28 $${k}_{r}=\frac{1}{{(1+r)}^{n}}$$

(c) Ultra-Capacitor System: The ultra-capacitor system's total annualized cost includes both initial installation and ongoing maintenance and operating costs.

29 $${TAC}_{inv}={{E}_{UCn}\times (C}_{cap}^{UC}\times CRF+{C}_{O\&M}^{UC})$$

In this instance, ${E}_{UCn}$ denotes the nominal capacity, measured in kilowatt-hours (kWh), of the ultracapacitor system. ${C}_{cap}^{UC}$ refers to the initial cost of the ultracapacitor, whereas ${C}_{O\&M}^{UC}$ indicates the expenses associated with its maintenance and operation.

(d) PHES System: The total annualized cost of the PHES system includes not only its initial investment but also its fixed and variable operating and maintenance cost. It is computed using the Eq. (30).

30 $${TAC}_{PHES}={(P}_{PHSn}\times {C}_{cap}^{PHES}+{E}_{c}\times {C}_{cap}^{stor})\times CRF +{P}_{PHSn}\times {C}_{O\&M-f}^{PHES}+{E}_{PHESn}\times {C}_{O\&M-V}^{PHES}$$

Here, the total annualized cost of the PHES over the considered period takes into account the total installed capacity ${P}_{PHSn}$ in kW, the initial installation cost ${C}_{cap}^{PHES}$ in €/kW, the total capacity of the upper reservoir in kWh and its construction cost ${C}_{cap}^{stor}$ in €/kWh, the fixed maintenance cost related to the total installed capacity and its specific cost ${C}_{O\&M-f}^{PHES}$ in €/MW and finally, the variable maintenance cost depending on the quantity ${E}_{PHESn}$ of energy produced by the PHES system over a year and its unit cost ${C}_{O\&M-V}^{PHES}$ in €/MWh.

#### Levelized cost of energy (LCOE)

The levelized cost of energy is used to calculate the lifetime cost of producing energy from a power plant. It is determined by^73^:

31 $$\text{LCOE} = \frac{{I}_{t}+{\sum }_{i=1}^{N}({O}_{t}+{M}_{t}/{(1+r)}^{i})}{{\sum }_{i=1}^{N}\left(\frac{{E}_{t}}{{\left(1+r\right)}^{i}}\right)}$$

where the variable ${I}_{t}$ denotes the capital investments required for the installation of renewable energy systems. The variable ${O}_{t}$ reflects the ongoing operational costs, while the variable ${M}_{t}$ indicates the expenses associated with maintenance;${E}_{t}$ is the annual energy produced.

#### Net present value (NPV)

The criteria for measuring a project’s cash flow were implemented to evaluate the profitability and payback time of the proposed new systems. The following equation expresses the NPV^74^:

32 $$NPV={\sum }_{i=1}^{N}\frac{{C}_{t}}{{\left(1+r\right)}^{i}}$$

where ${C}_{t}$ is the difference between cash inflow and outflow.

## Formulation of problems and optimization

The optimization problem in this study is bi-objective and is represented by Eq. (33). Equation (34) describes the constraints for this multi-objective optimization problem.

33 $${\text{min}}(Objective\, Functions)=min\left\{\begin{array}{l}Reliability\, Parameter \left\{LPSP\right\}\\ Economical\, Parameter \left\{TAC\right\}\end{array}\right.$$

34 $$\left\{\begin{array}{l}{N}_{PV}^{L}\le {N}_{PV}\le {N}_{PV}^{U}\\ {E}_{UC}^{L}\le {E}_{UC}^{max}\le {E}_{UC}^{U}\\ {P}_{PHSn}^{L}\le {P}_{PHSn}\le {P}_{PHSn}^{U}\\ {V}_{n}^{L}\le {V}_{max}\le {V}_{n}^{U}\end{array}\right.$$

where, ${N}_{PV}^{L}$, ${P}_{PHSn}^{L}$, ${V}_{n}^{L}$, ${E}_{UC}^{L}$, represent the lower bounds of the optimization variables. ${N}_{PV}^{U}$, ${P}_{PHSn}^{U}$, ${V}_{n}^{U}$, ${E}_{UC}^{U}$ represent the upper bounds of the optimization variables.

## Optimization algorithms

In this paper, the optimization problem was solved using six different optimization techniques. These include multi-objective bonobo optimizer (MOBO), multi-objective salp swarm algorithm (MSSA), multi-objective ant lion optimizer (MOALO), multi-objective particle swarm optimization(MOPSO), improving the strength pareto evolutionary algorithm (SPEA2)and multi-objective African vulture’s optimization algorithm (MOAVOA).

MOBO is an adaptation of the bonobo optimizer(BO)technique introduced in Ref.^75^ that is designed to address multi-objective challenges. This algorithm takes its inspiration from the social behavior of bonobos^76^. Several variants of this method are available, including a version that integrates a distance sorting approach and non-dominated crowding (MOBO-II)^77^. This algorithm has been employed to address a wide range of optimization issues, particularly as documented in references^78–82^. The variation employed in this work is the proposed one, which incorporates distance sorting and a non-dominated crowding approach.

MSSA is a multi-objective problem-solving version of the salp swarm algorithm(SSA) approach developed in^83^. It is an algorithm inspired by the swarming and feeding behavior of salp in the seas. This approach has been used for a wide range of optimization problems, most notably in^84–87^. This method was especially intended to handle the challenge of sizing renewable energy plants^88–91^.

MOALO is an adaptation of the ant lion optimizer(ALO) for multi-objective problem solving^92^. It is a natural-inspired algorithm, more especially the hunting techniques used by ant lions^93^. This approach has been used for a wide range of optimization problems, particularly in Refs.^94–96^.

The MOAVOA metaheuristic, which was introduced in Ref.^97^, is an adaptation of the African vultures optimization algorithm (AVOA) technique^98^ designed to solve multi-objective problems. It is an algorithm inspired by nature, specifically the navigation and foraging behaviors of African vultures. Numerous optimization problems have been resolved utilizing this algorithm, including those in Refs.^99,100^.

MOPSO is the multi-objective problem-solving version of the PSO technique^101^. This algorithm has been used for the dimensioning of renewable energy systems. It is one of the most popular algorithms^102–104^.

SPEA2, an improvement on SPEA^105^, is also a proven algorithm for solving complex optimization problems^106,107^.

## Proposed system operational strategies

Energy flow management on the grid is critical for increased grid resilience.

If ${P}_{grid}\left(t\right)>{P}_{grid}^{max}$, the PV system output will be reduced until ${P}_{grid}$ equals ${P}_{grid}^{max}$.

If ${P}_{grid}\left(t\right)\le {P}_{grid}^{max}$, the electrical grid's power flow is managed as follows:

Three situations are considered:

I. If,${{\eta }_{inv}\times P}_{PV}(t)={P}_{DT}(t)$, the output of the PV field equals the demand.
II. If,${{\eta }_{inv}\times P}_{PV}\left(t\right)>{P}_{DT}(t)$, the PV field output exceeds the demand. The extra energy is used to charge the two storage systems, and the remainder is deemed excess energy.
III. If,${{\eta }_{inv}\times P}_{PV}\left(t\right)<{P}_{DT}(t)$, PV field output is insufficient to fulfill demand. In this scenario, if the PHES plant were turned off, the ultra-capacitor system would provide the energy required to fulfill demand over a three-minute period. Following that, the PHES power plant takes over and meets the long-term energy needs.

**Figure 5:**
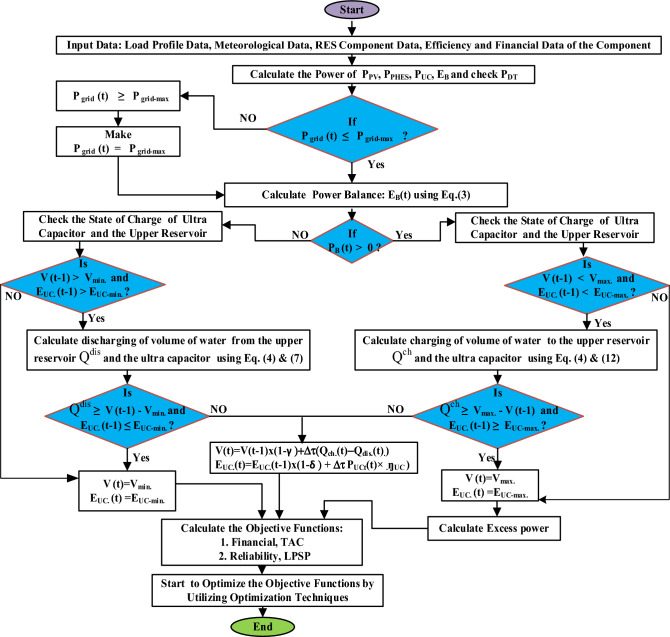
Flowchart describing the operation of the proposed grid-connected solar PV/PHES/Ultra-capacitor system.

The flow chart in Fig. 5 shows the proposed energy flow management strategy for new renewable energy systems.

## Result and discussion

**Figure 6:**
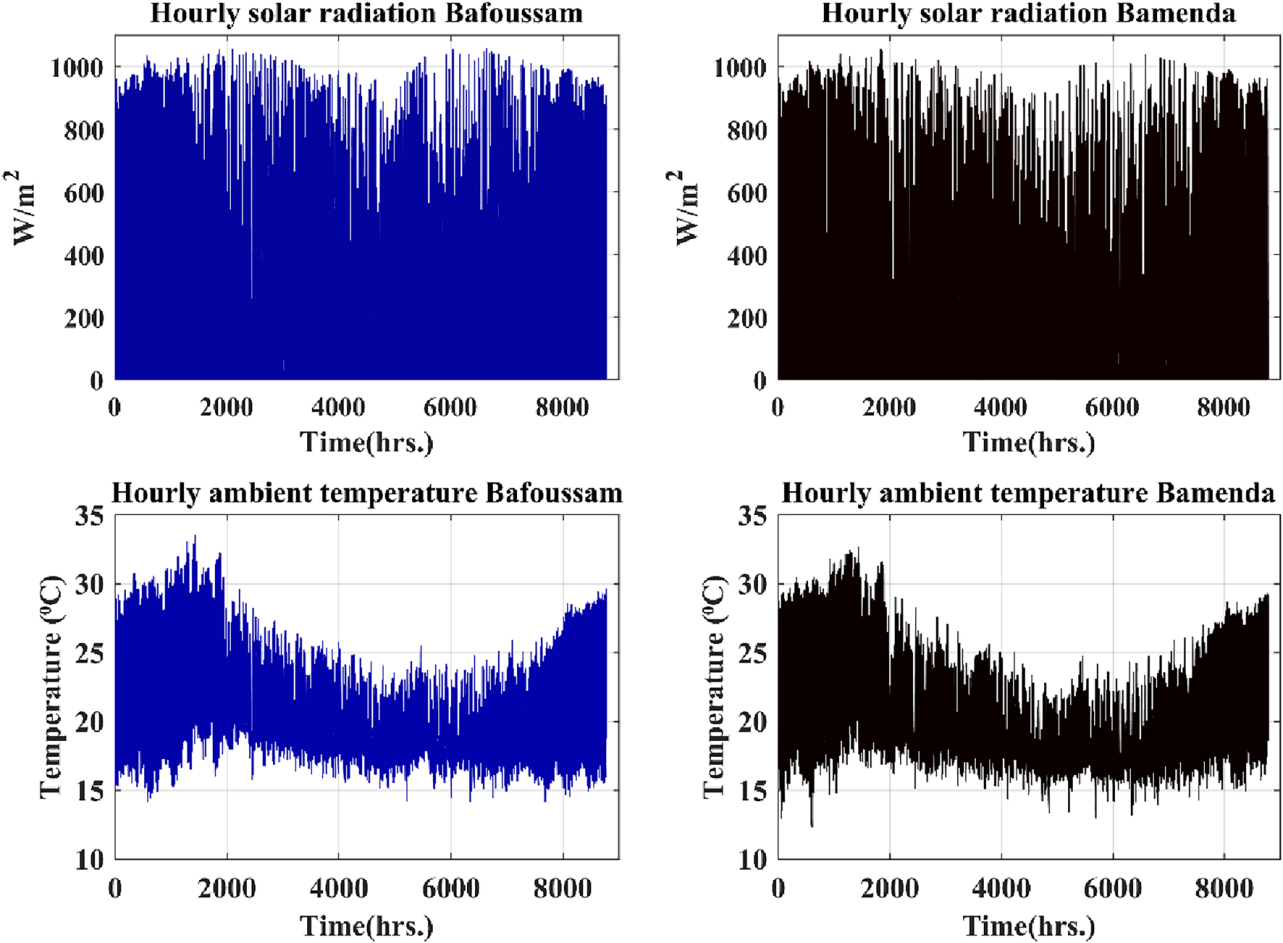
Bafoussam and Bamenda hourly solar radiation and temperature data over a year.

**Figure 7:**
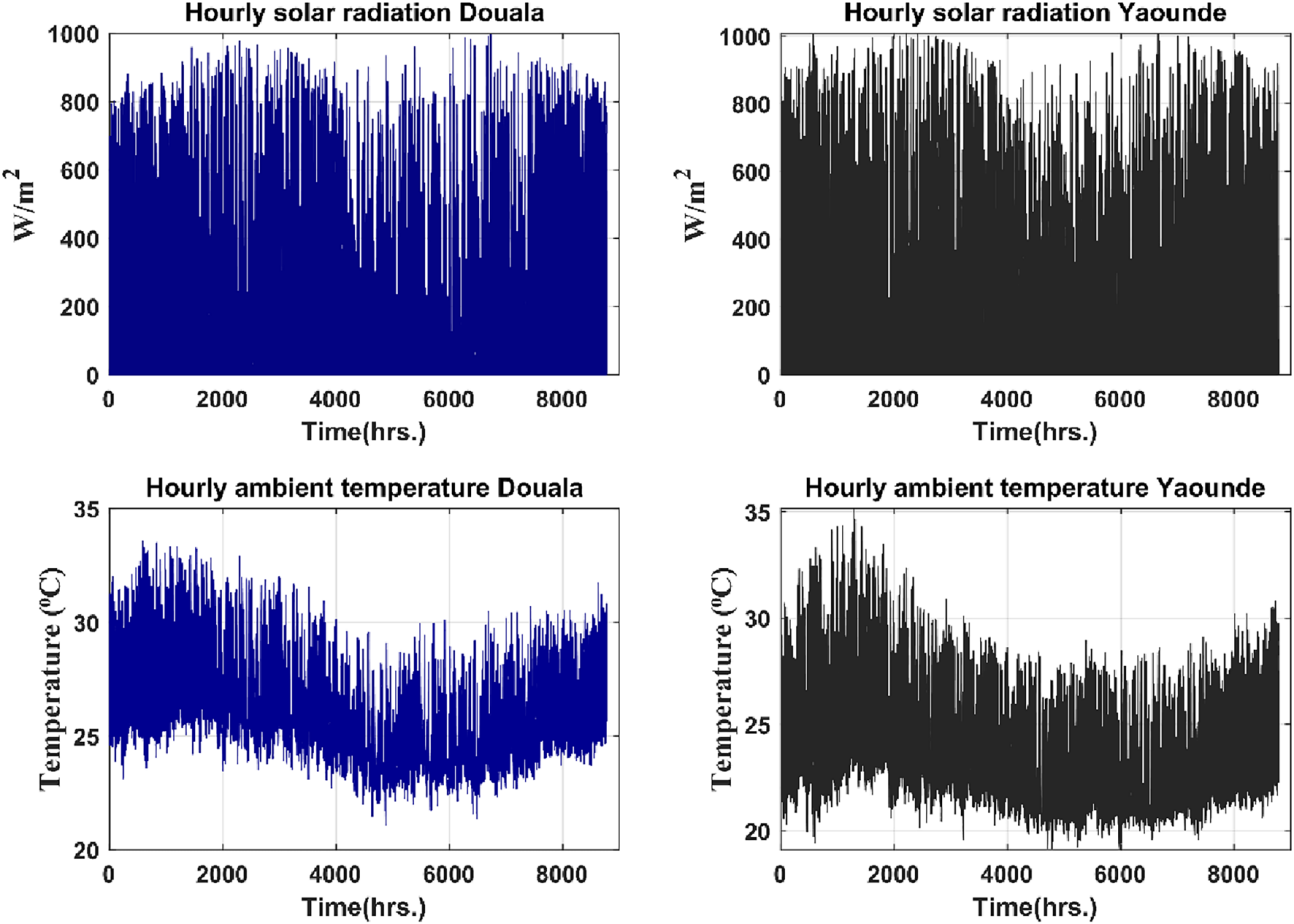
Douala and Yaounde hourly irradiation and temperature data over a year.

**Figure 8:**
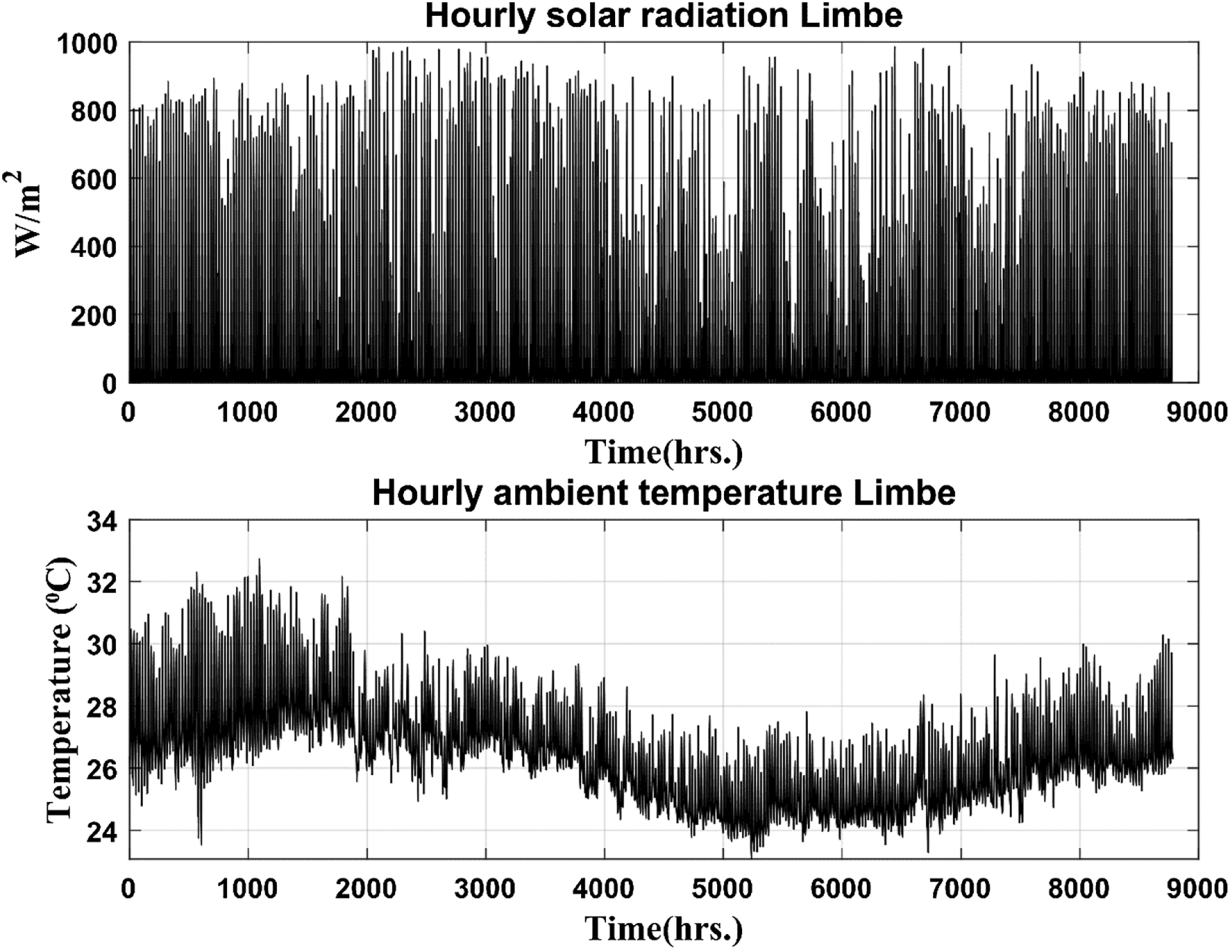
Limbe hourly irradiation and temperature data over a year.

Figures 6, 7 and 8 show the hourly meteorological data for the locations selected as potential sites for the PV solar field. The meteorological data were acquired from^108^.

### Optimization algorithm results

**Figure 9:**
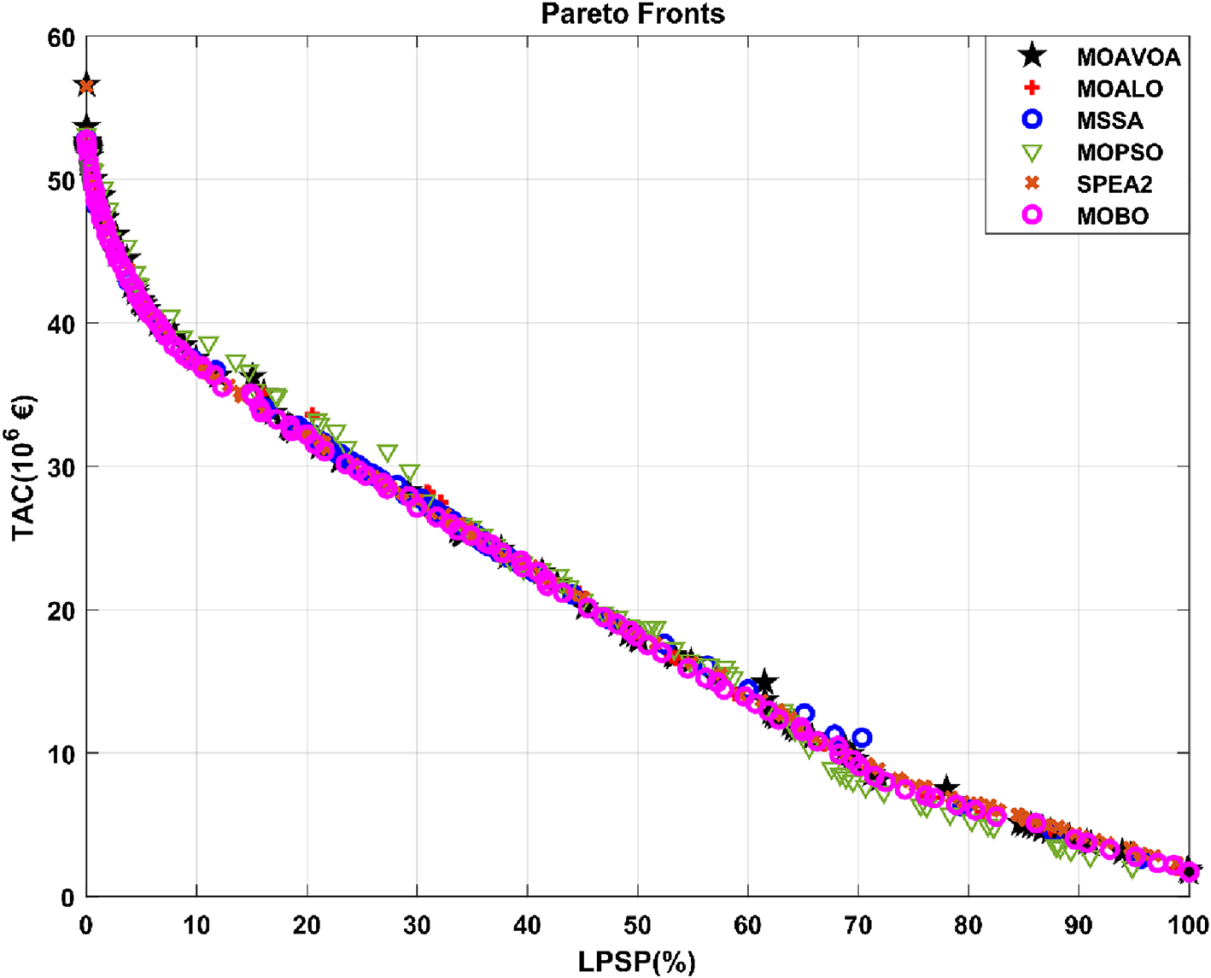
Pareto fronts obtained with different algorithms.

The locality of Bafoussam was first chosen for the installation of the solar PV field in order to evaluate the performance of each optimization algorithm. The Pareto fronts obtained through the utilization of the MOBO, MSSA, MOALO, SPEA2, MOPSO, and MOAVOA algorithms are depicted in Fig. 9.

Figure 9 reveals several key points. All six curves generated by the six algorithms are the same shape. When the criterion for measuring load shedding rate (LPSP) approaches zero, the total annualized project cost (TAC) for the MOBO, MSSA, MOALO, MOAOA, MOPSO, and SPEA2 meta-heuristics rises to 52.78 × 10^6^ €, 52.87 × 10^6^ €, 56.61 × 10^6^ €, 53.08 × 10^6^ €, 53.17 × 106 €, and 56.49 × 10^6^ €, respectively. The second most obvious visual observation in Fig. 9 is that the six Pareto fronts generated by the six metaheuristics are nearly identical. The curves of the Pareto fronts show the contradictions between the objectives. TAC increases while LPSP decreases. It is worth noting that all points on the Pareto fronts provide optimal solutions for the specified configurations. These configurations correspond to the sizes of the renewable energy systems that will be installed, including PV, ultra-capacitor, and PHES capacities. Because the goal of this work is to completely replace LFO and HFO thermal power plants, the remainder of the analysis will concentrate on the results obtained for the lowest LPSPs from each algorithm. The LPSP criterion here assesses the dependability of new renewable energy systems. When LPSP is zero, system reliability is 100%; when LPSP is 100%, then system reliability is zero. Table 1 displays the results of the six algorithms.

**Table 1:** The results provided by the six optimization algorithms for LPSP equal to zero after simulation.

Algorithm	LPSP (%)	TAC (10^6^€)	PV (MW)	PHES (MW)	Storage (m^3^)	Euc (MWh)
MOBO2	0	52.78	350.06	182.64	1,252,768.10	10
MSSA	0.000078	52.87	346.66	193.30	1,246,216.85	10.06
MOAVOA	0	56.61	390.84	154.98	1,327,083.69	12.86
MOALO	0.0039	53.08	348.97	178.76	1,277,494.60	12.50
MOPSO	0	53.17	357.51	168.10	1,268,342.25	11
SPEA2	0	56.49	389.20	156.23	1,367,540.21	11.10

After analyzing the data in Table 1, the MOBO algorithm gave the most satisfactory result in terms of total annualized costs. The simulation results indicate that the total annualized costs of the MOBO, MSSA, MOALO, MOAVOA, MOPSO, and SPEA2 meta-heuristics are 52.78 × 10^6^ €, 52.87 × 10^6^ €, 53.08 × 10^6^ €, 56.61 × 10^6^ €, 53.17 × 10^6^ €, and 56.49 × 10^6^ €, respectively. The TAC achieved using the MOBO algorithm is 0.17%, 0.57%, 0.73%, 7.03%, and 7.26% lower compared to the TACs achieved using the MSSA, MOALO, MOPSO, SPEA2, and MOAVOA algorithms, respectively. The reported TACs for the metaheuristics MOBO, MOPSO, SPEA2, and MOAVOA are zero for LPSPs. However, the TACs for the metaheuristics MSSA and MOALO are 0.000078% and 0.0039%, respectively. As a result, compared to the other five methods, the TAC derived from the MOBO algorithm performs better. The consistent outcomes of the six algorithms serve as evidence of their exceptional quality and mutually validate the results they generate. MOBO was selected to analyze the influence of different reliability criteria on TAC and the optimal placement of PV system installation based on its superior performance compared to the other five algorithms. Table 1 also displays the various algorithm arrangements, such as the capacity of the solar, ultra-capacitor, and PHES systems.

### Impact of reliability criteria on TAC

**Figure 10:**
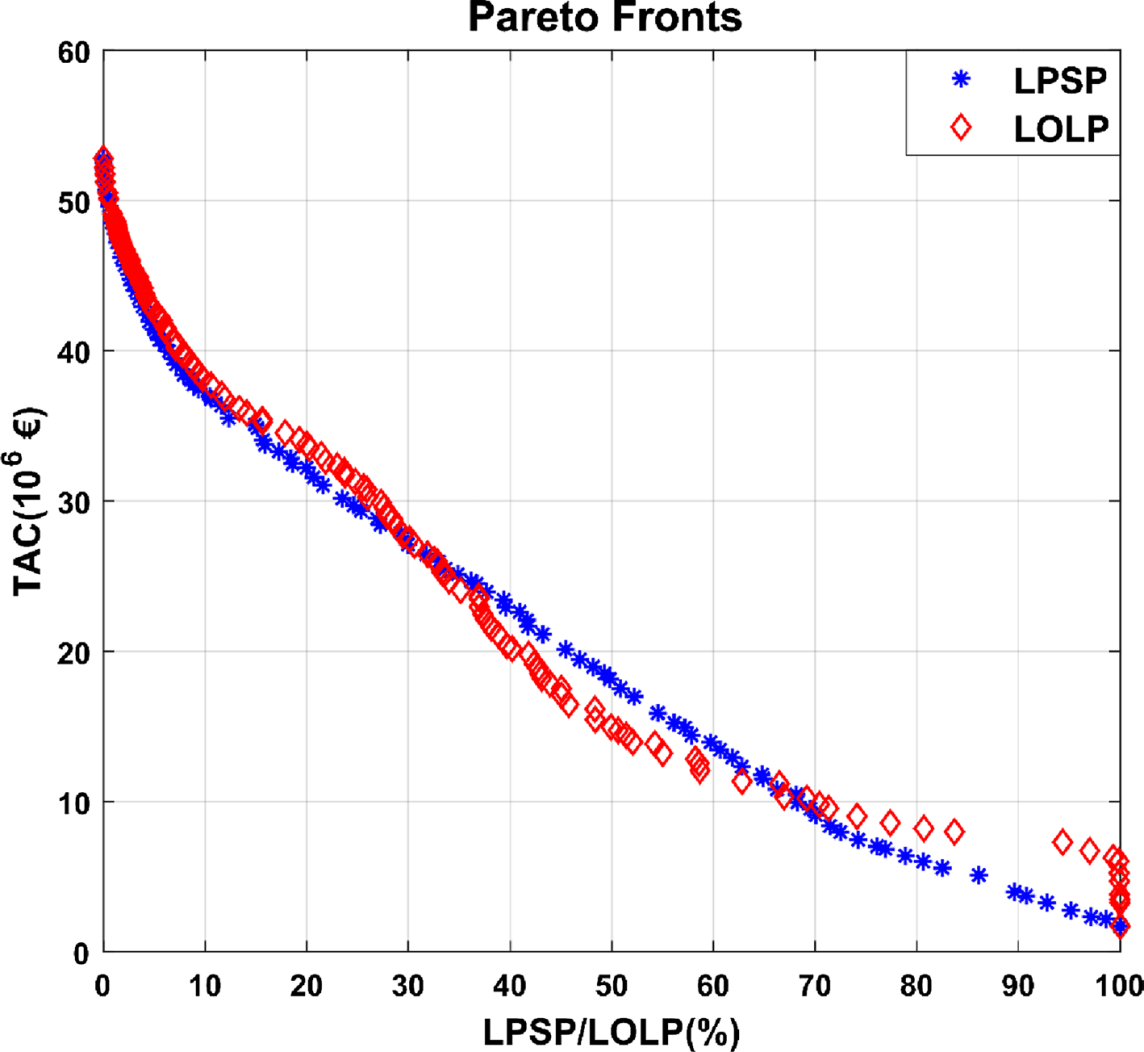
Pareto fronts obtained for LPSP/LOLP.

Figure 10 depicts the Pareto fronts obtained in this study for the two reliability criteria assessed for new renewable energy systems.

In Fig. 10, the Pareto fronts obtained for the two-reliability criterion have comparable curves. Total annualized cost rises when LOLP and LPSP decreases. As LPSP and LOLP approach 0, the Pareto front curves converge. When the LPSP/LOLP is equal to zero, the TACs estimated for the two reliability criteria are nearly identical, as shown in Table 2. The differences between the two Pareto fronts in Fig. 10 are primarily due to the LOLP reliability criterion, which considers the time when renewable energy systems were able to meet the entire demand. When the energy available at a given time can only meet a portion of the demand, the LOLP criterion classifies this as a time when the renewable energy system was unable to meet the load.

**Table 2:** Effects of LPSP & LOLP on TAC.

Criteria	TAC (€)	PV (MW)	PHES (MW)	Storage (m^3^)	Euc (MWh)
LPSP	52.78 × 10^6^	350.06	182.64	1,252,768.85	10
LOLP	52.80 × 10^6^	354.52	182.83	1,188,565.20	10

### Impact of the location of PV system on TAC

**Figure 11:**
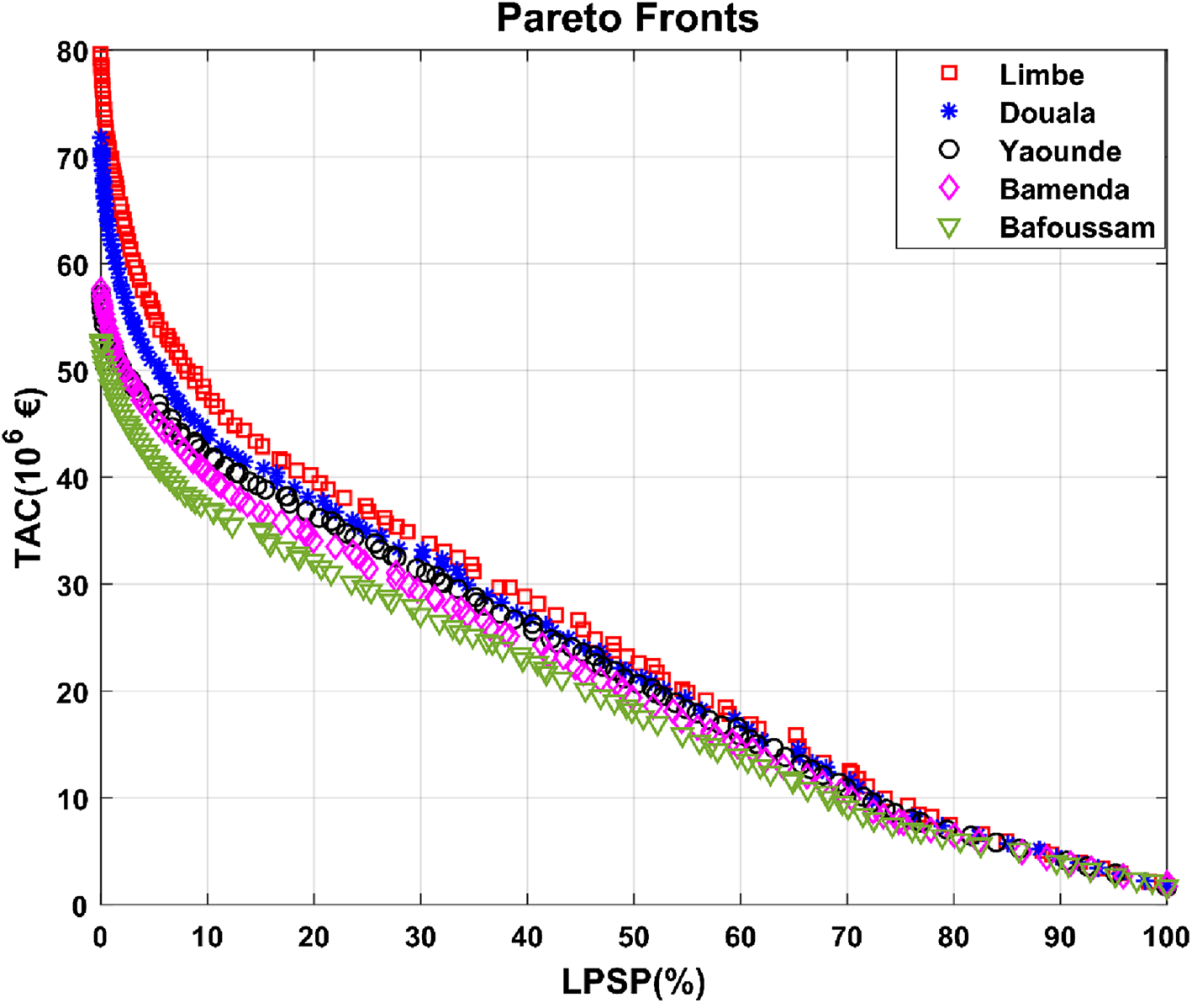
Pareto fronts obtained for Bafoussam, Bamenda, Yaoundé, Douala and Limbe.

The prospective sites for the solar plant were selected based on the geographical coverage of the southern connected grid and the locations of the thermal power facilities. The outcomes acquired in this instance are displayed in Fig. 11.

Figure 11 shows that among the other locations, Bafoussam had the lowest total yearly cost of PV installation. It is also worth noting that Bamenda and Yaoundé had economically interesting outcomes. The coastal towns, on the other hand, provided the least substantial economic gains. Limbe had the poorest TAC results. The substantial variance in TACs discovered for different places is mostly explained by the large variation in PV system capacity depending on the locality under consideration. According to Table 3, the PV system size determined for Bafoussam is 350.06 MW, compared to Limbe's 574.78 MW. As a result, the PV system planned for Limbe is 1.64 times larger than the PV system planned for Bafoussam. The same is true for the other towns. The size of the PV system is determined by the amount of solar radiation received at each site. As a result, installing a PV field in Bafoussam is more cost effective than in the other towns considered. Yaoundé, in addition to Bafoussam, will be included in the detailed economic assessments due to its administrative, demographic, and geographical significance.

**Table 3:** Impact of PV field installation site on TAC (LPSP = 0%).

Locations	TAC (€)	PV (MW)	Pphs (MW)	Storage (m^3^)	Euc (MWh)
Bafoussam	52.78 × 10^6^	350.06	182.64	1,252,768.85	10
Yaoundé	57.07 × 10^6^	390.81	167.30	1,477,515.22	10
Bamenda	57.57 × 10^6^	393.12	200	1,241,794.98	10
Douala	71.82 × 10^6^	520.10	199.99	1,628,329.03	10
Limbe	79.63 × 10^6^	574.98	200	2,000,000.00	10

**Figure 12:**
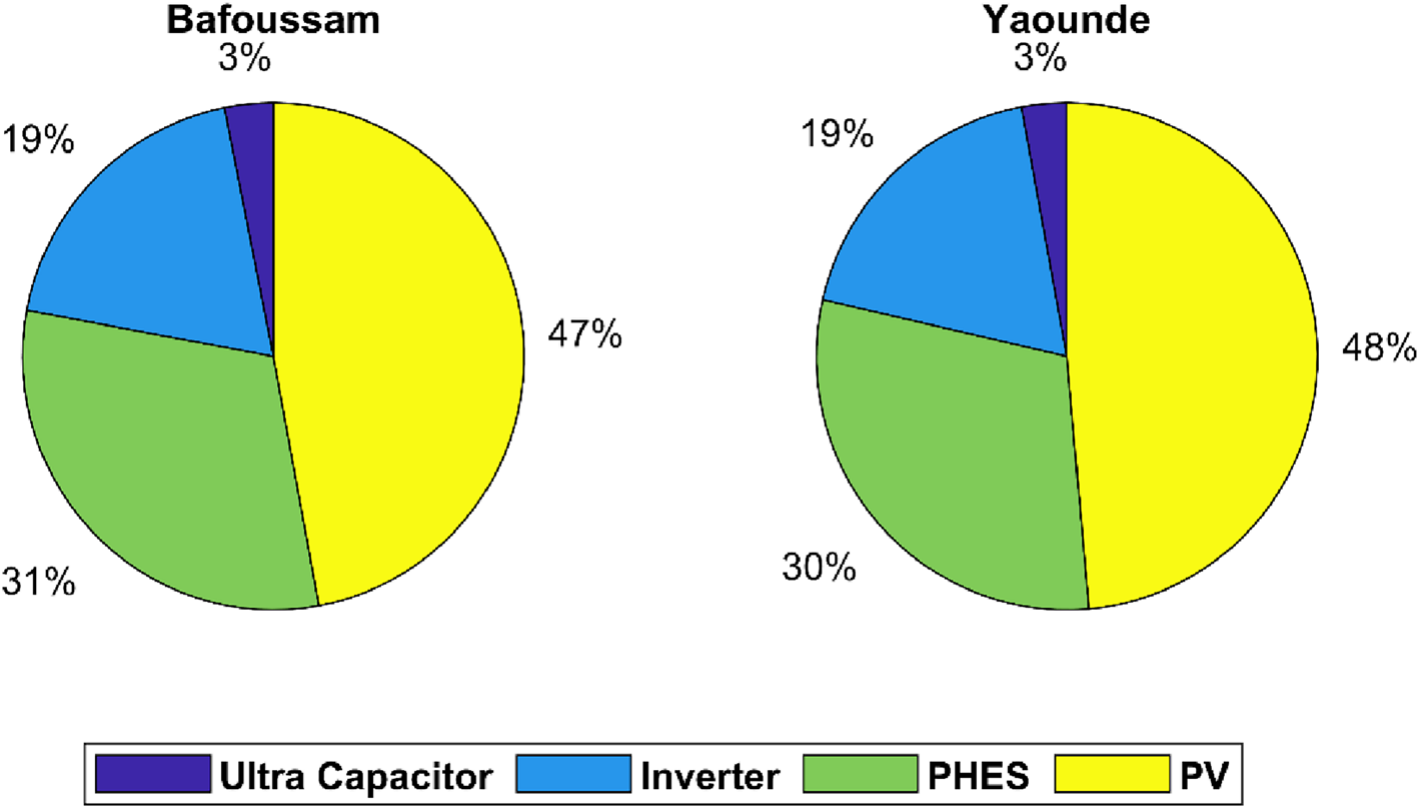
Share of each component in the project's TAC.

Figure 12 depicts the cost-sharing arrangements of renewable energy system components.

**Figure 13:**
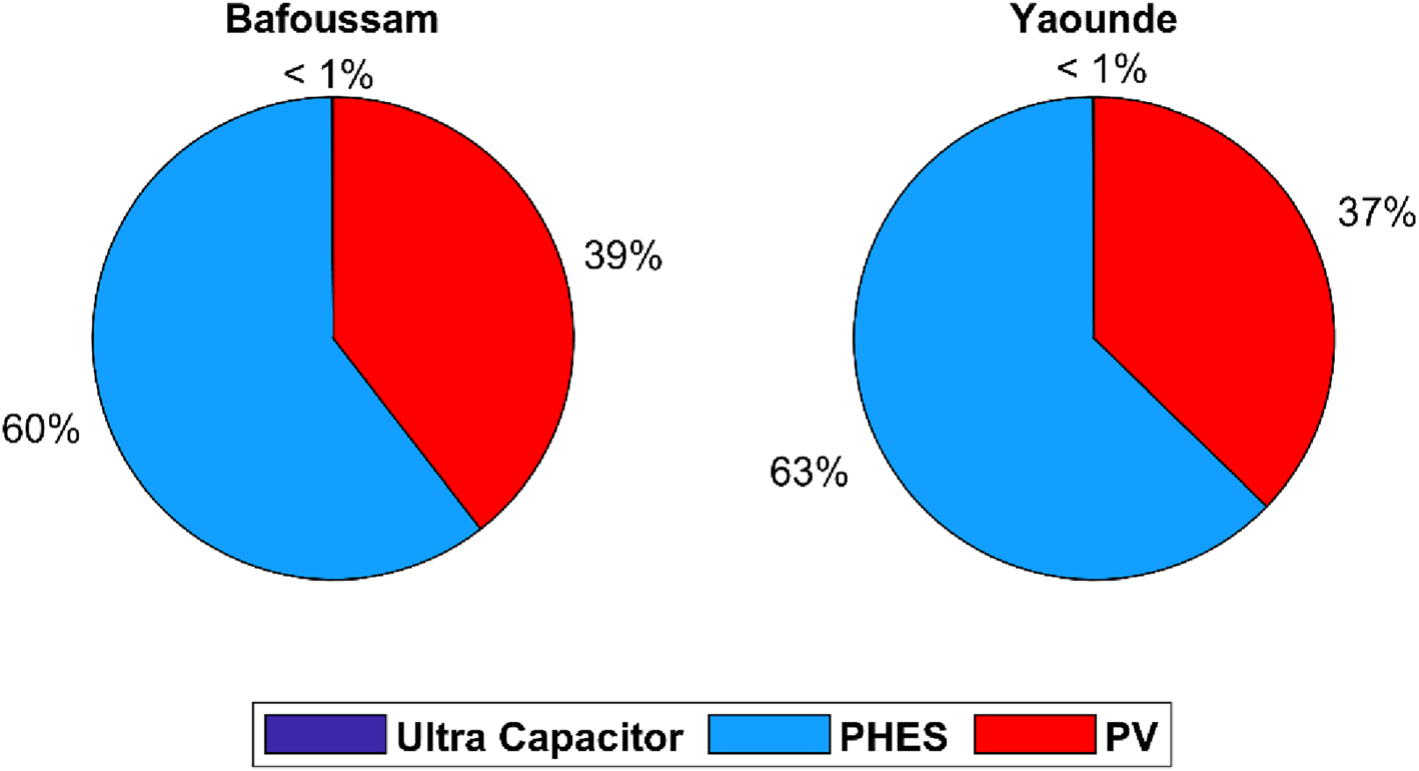
The role of storage in meeting total energy demand.

Figure 12 depicts the PV system accounting for 47% of the total annualized project cost in the case where the PV system installation location is Bafoussam, the PHES system accounting for 31%, inverters accounting for 19%, and ultra-capacitors accounting for the remaining 3%. In the case of a PV system installed in Yaoundé, the PV system accounted for 48% of the total annualized cost of the project, the PHES system for 30%, inverters for 19%, and ultra-capacitors for the remaining 3%. In the two cases investigated, photovoltaic systems account for a more significant percentage of expenses than PHES storage systems. The contribution of each renewable energy source and storage technology to meeting demand is depicted in Fig. 13.

**Figure 14:**
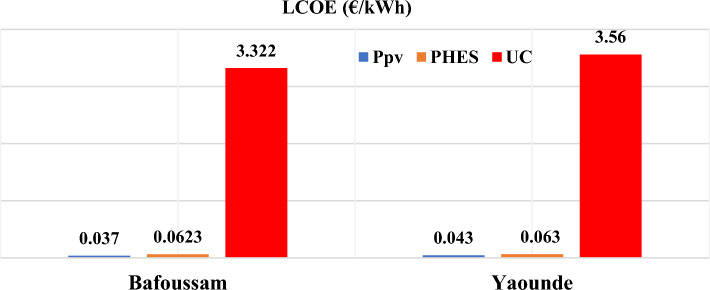
Levelized cost of energy obtained for Bafoussam and Yaounde.

In any event, ultra-capacitor systems provide very little energy compared to PHES and PV systems. The PHES system supplied 60% of the energy to the loads in the Bafoussam case, while the PV system supplied the remaining 39%. In the Yaoundé case, the PHES system supplied 63% of the required annual load, while the PV system supplied 37%. Figure 14 depicts the LCOEs for the PV, PHES, and ultra-capacitor systems to assess the proposed systems' competitiveness.

The LCOE for the PV, PHES, and ultra-capacitor systems in the Bafoussam case is 0.037€/kWh, 0.0623€/kWh, and 3.322€/kWh, respectively, according to Fig. 14. The LCOE for the PV, PHES, and ultra-capacitor systems in the Yaoundé scenario is 0.043€/kWh, 0.063€/kWh, and 3.56€/kWh, respectively. The average cost of energy supplied by ultra-capacitors is quite high, owing to their position as a fast-response storage system, but also to the fact that their share of the overall system cost in any scenario is 3%, despite the fact that their total yearly energy supplied is less than 1% of demand.

**Figure 15:**
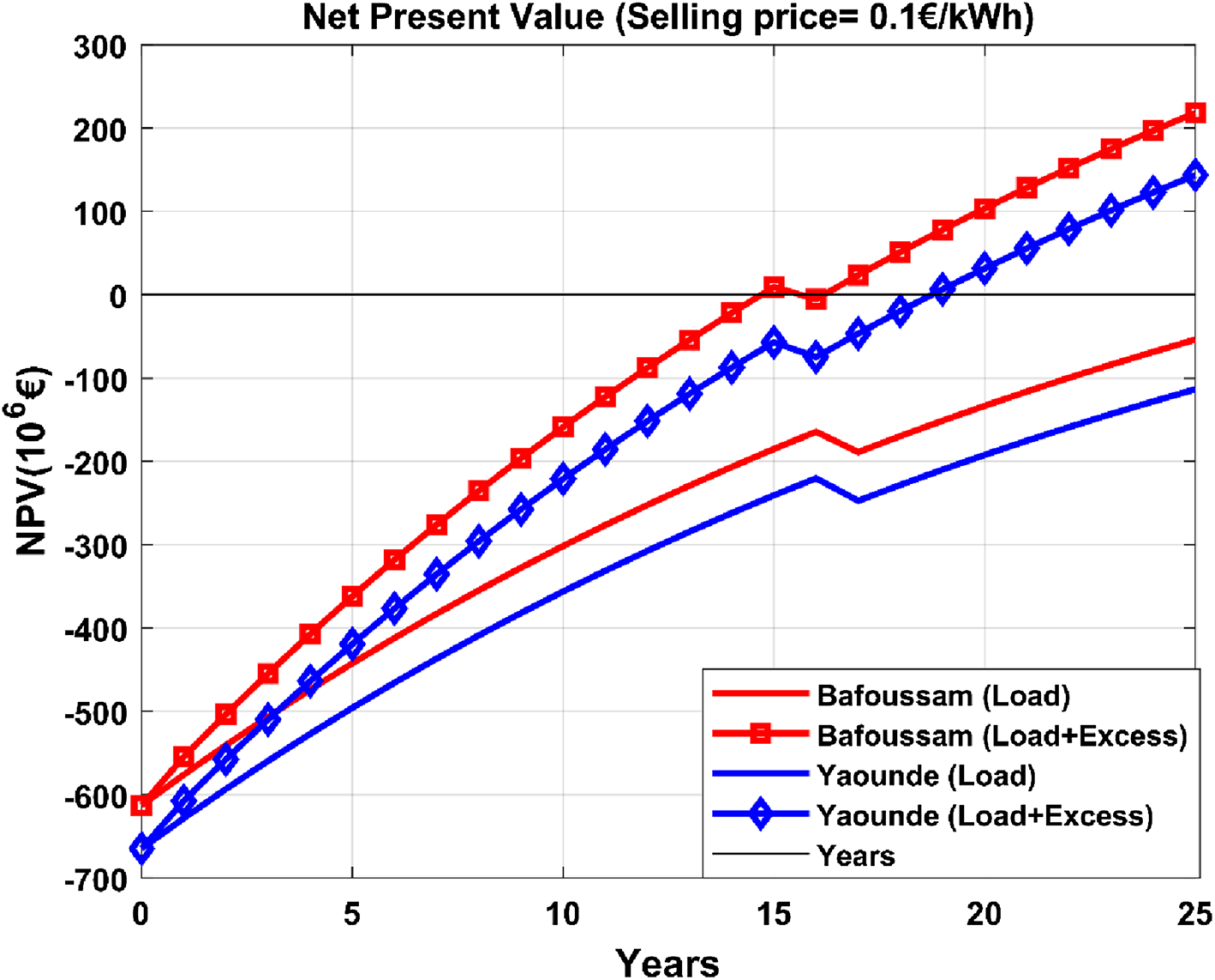
Net present value for an energy selling price of 0.10€/kWh.

**Figure 16:**
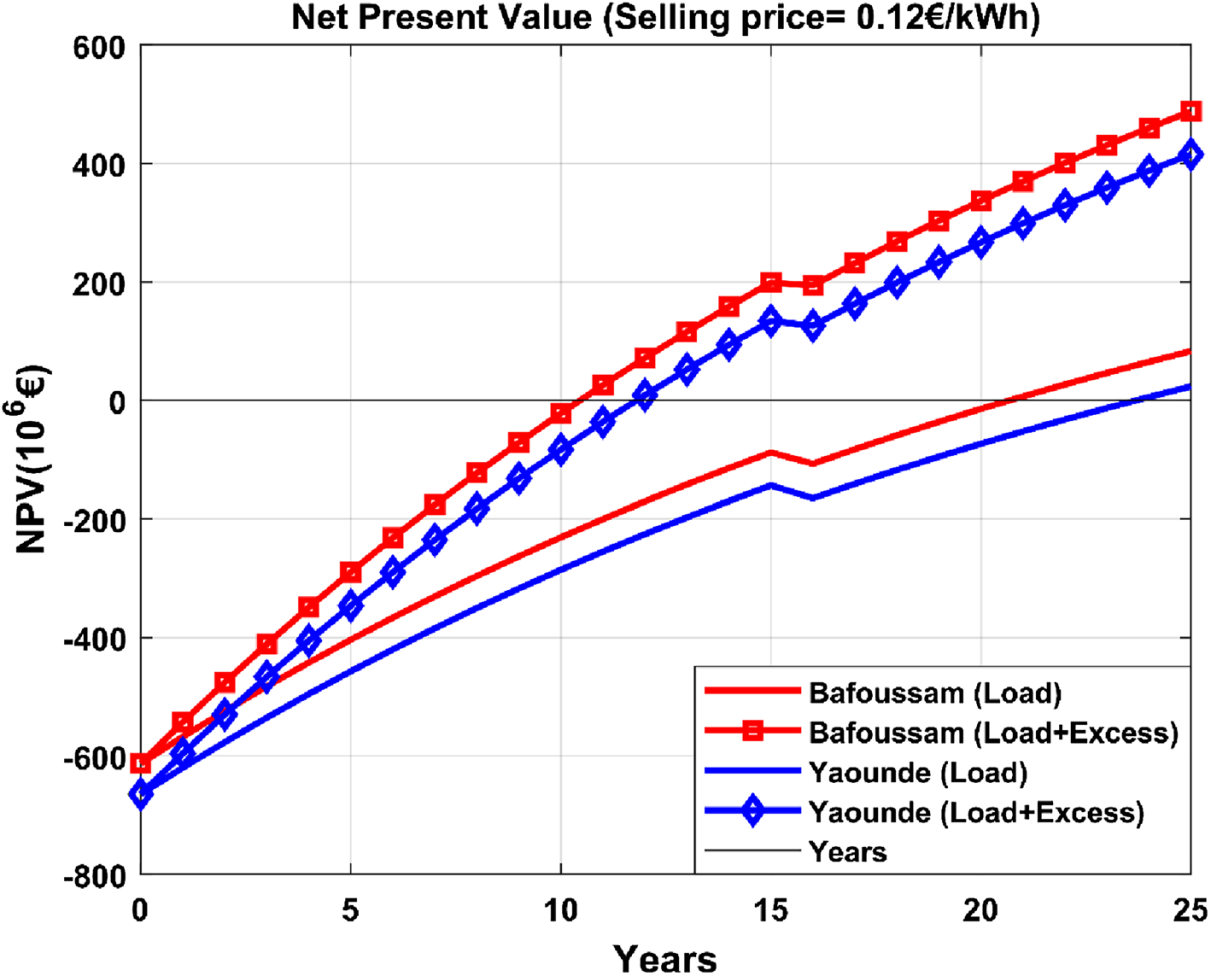
Net present value for an energy selling price of 0.12€/kWh.

Figures 15 and 16 represent the net present value over time to analyze the project's payback period.

Figure 15 illustrates that regardless of the chosen site for the PV system installation, the investment does not become profitable until the end of the project's lifespan if only load demand is considered. The Bafoussam and Yaoundé scenarios become cost-effective when surplus energy is considered, with payback periods of 17 and 19 years, respectively.The energy-selling price was reassessed to determine its impact on the project's profitability. Figure 16 depicts the consequences of increasing the selling price from 0.1€/kWh to 0.12€/kWh.

Figure 16 demonstrates that the project is profitable under all scenarios analyzed. The payback periods for the Bafoussam and Yaoundé scenarios are 21 and 24 years, respectively, when considering only load demand. When surplus energy is absorbed and utilized by the power grid, payback periods are significantly shortened. The payback periods for Bafoussam and Yaoundé are 11 and 12 years, respectively, when excess energy is taken into account. The project's profitability is mainly determined by the energy sales price. The project becomes profitable when electricity is sold at €0.12/kWh.

**Figure 17:**
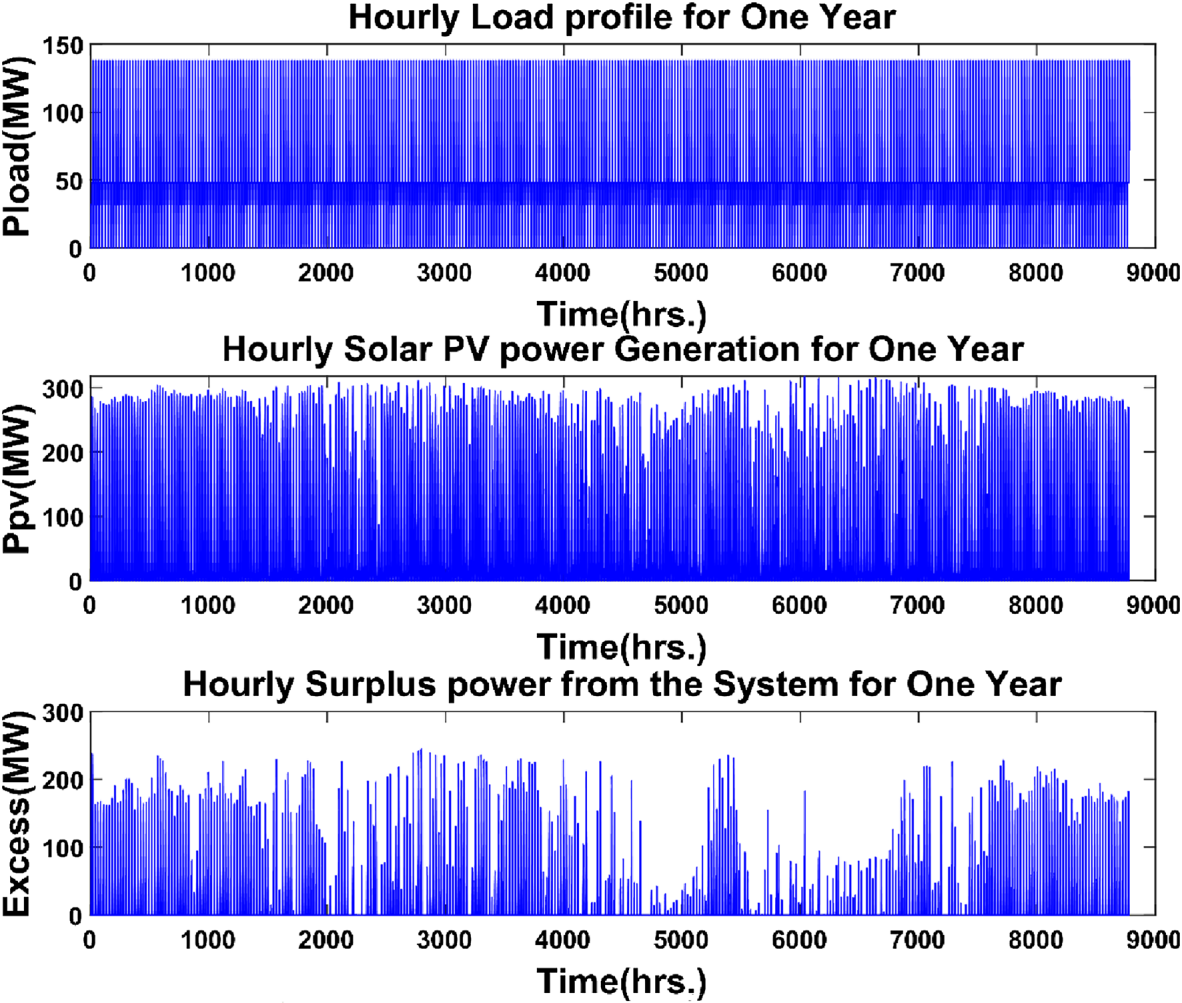
Hourly variations of load, solar PV power generation, and excess power on the grid.

The dynamic behavior of the energy production/consumption of the PV system, the PHES power plant, and the ultra-capacitor system over a one-year period was analyzed only for the results obtained for the Bafoussam locality. Figure 17 depicts the demand, PV system output, and extra energy produced for the Bafoussam case during a one-year timeframe.

According to Fig. 17, the photovoltaic field produced a maximum power of 318 MW and an annual energy generation of 671.84 GWh.The surplus energy produced can reach a maximum capacity of 237 MW at times, with an annual energy surplus of 153.022 GWh. The surplus energy accounts for approximately 22.78% of the annual energy produced by the photovoltaic system.Installing hydrogen production systems based on water electrolysis would be one method of using this extra energy. The hydrogen generated might then be sold.

**Figure 18:**
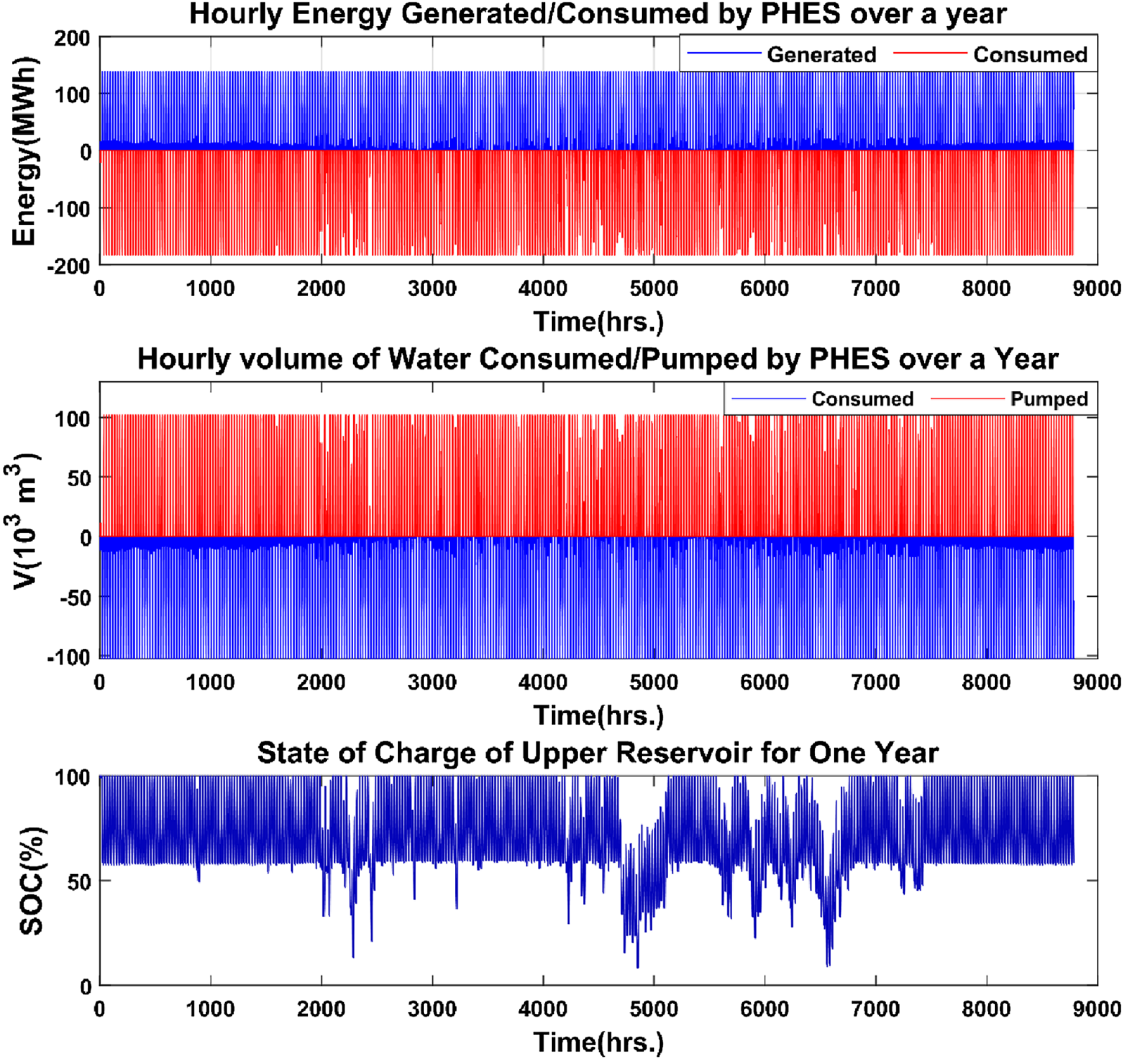
Energy produced/consumed and volume of water discharged/pumped by the PHES system.

Figure 18 displays the energy generated/consumed, the quantity of water consumed/pumped, and the state of charge in the upper reservoir of the PHES system.

**Figure 19:**
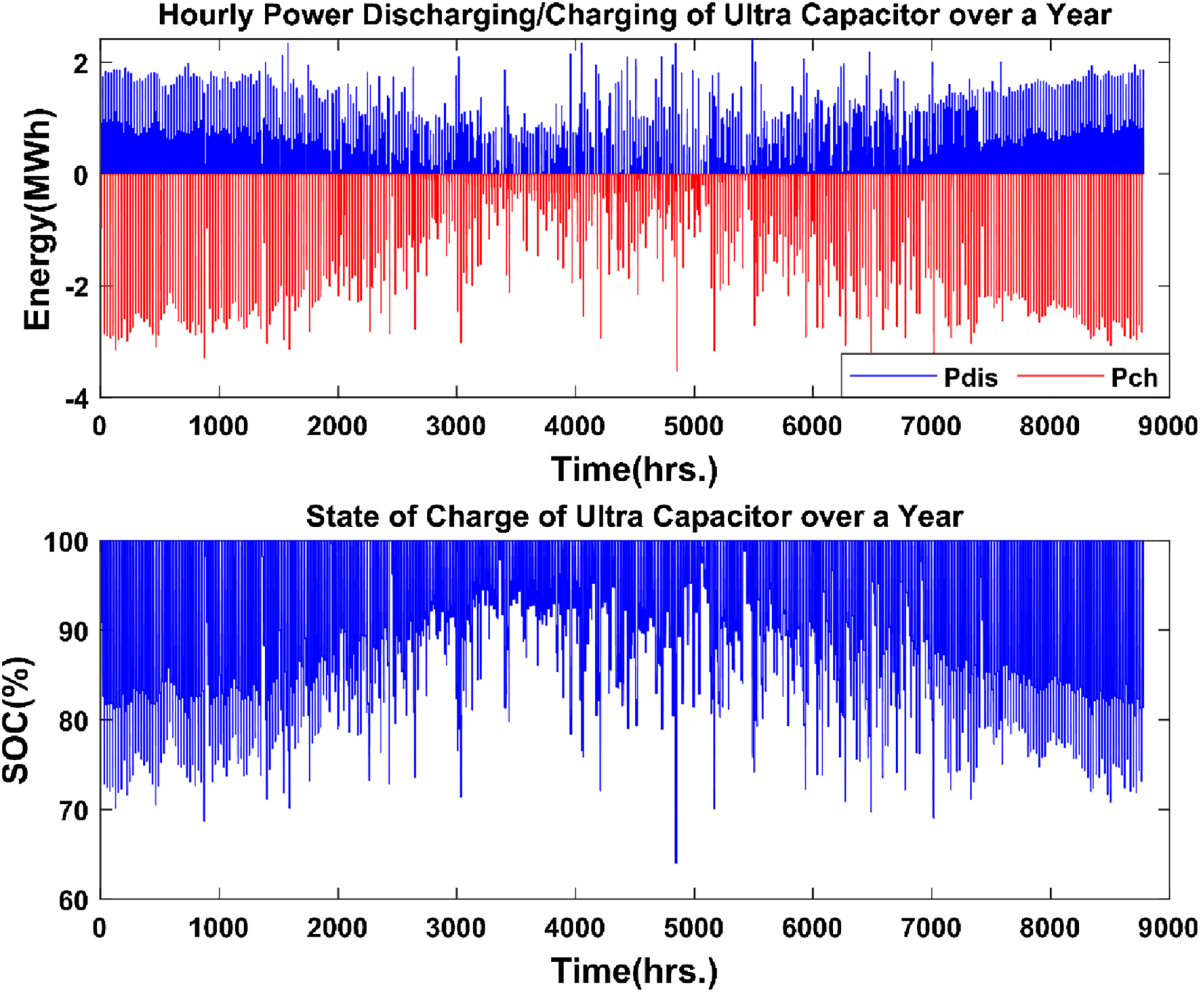
Ultra-capacitor charging/discharging power over a one-year period.

The yearly energy produced by the PHES system is 271.27 GWh, and the highest power output is 138 MW, which corresponds to the maximum load demand, as shown in Fig. 18. The yearly energy consumption in water pumping mode is 347.43 GWh, and the PHES system's maximum power in pumping mode is 182.64 MW, corresponding to the PHES system's rated capacity. The PHES system's energy generated to energy-consumed ratio is roughly 0.75, equating to the system's round-trip efficiency. The highest amount of water utilized to generate electricity is 102.50 thousand m^3^, which is equivalent to the amount of water necessary to generate 138 MW of power for a PHES system at a height of 560 m. The PHES system can pump a maximum of 101.74 thousand m^3^ of water into the higher reservoir. The state of charge of the upper reservoir strictly complies with the minimum water limit set for the PHES reservoir. Figure 19 depicts the discharge and charge energy of the ultra-capacitor system.

**Figure 20:**
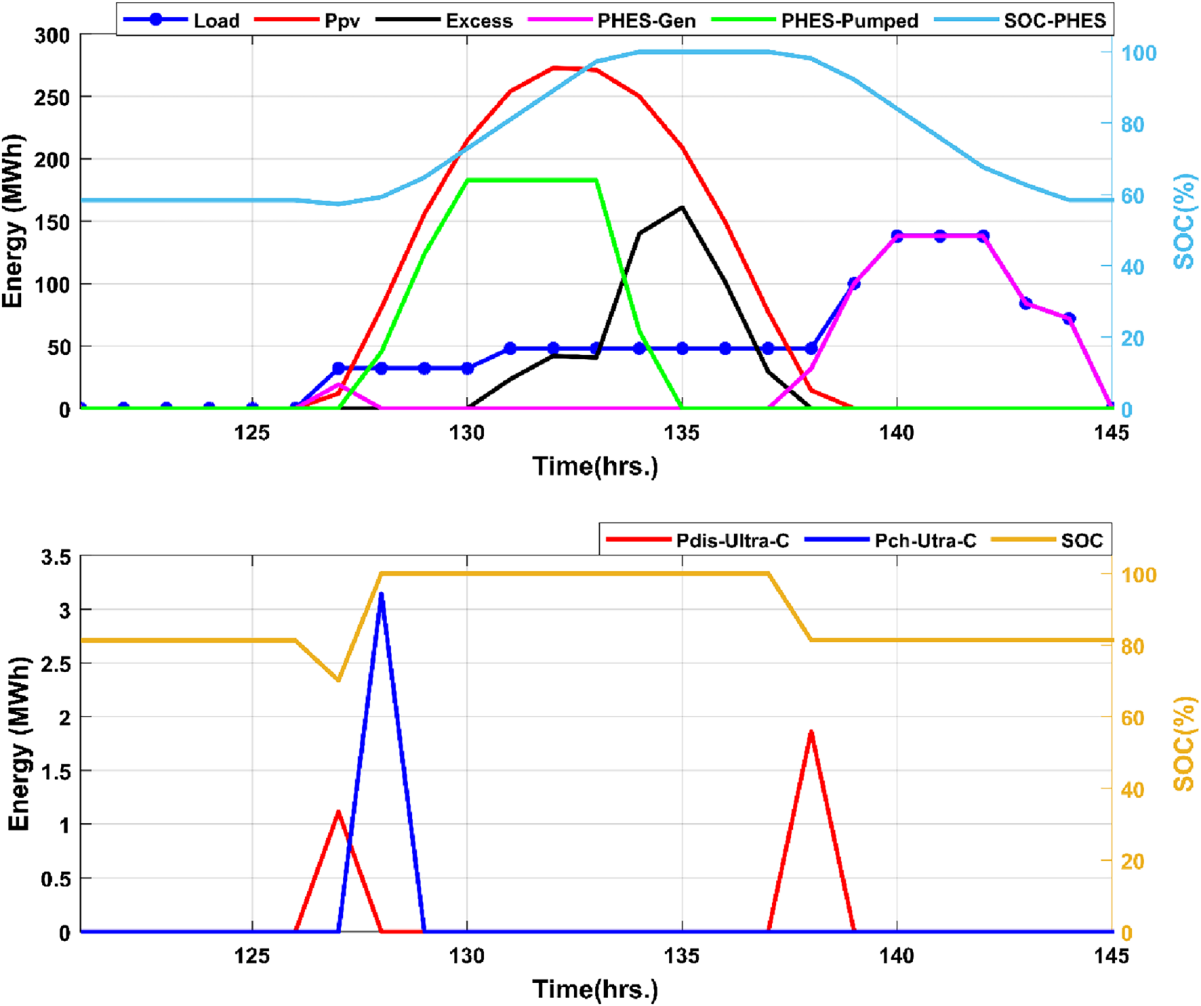
A typical one-full day 24-h operation.

The annual discharge energy generated by the ultracapacitor system is negligible in comparison to the energy produced by the PHES system, totaling 717.005 MWh, whereas the annual energy used to charge the ultracapacitor system is 752.78 MWh. Figure 20 depicts the dynamic behavior of the energy production curves over a 24-h period to help comprehend the operational and energy management processes for the overall renewable energy system.

According to the results of Fig. 20, the ultra-capacitor system discharges only during transitory periods, when the PHES system must begin producing energy. The discharge curve of the ultra-capacitor system is in energy magnitude and corresponds to the power provided by the ultra-capacitors throughout the 3-min period required to start the PHES system. It is also worth noting that the charging energy of the PHES system is limited by the PHES system's nominal capacity and the state of charge of the upper reservoirs. The ultra-capacitor system's function in power system reliability is critical. Fast-response storage systems will play an increasingly important role in grid stability as intermittent renewable energy technologies are increasingly integrated into power networks.

## Conclusion and future research directions

This study investigated the size of renewable energy systems comprised of a PV field, a PHES power plant, and an ultra-capacitor system for quick response to demand. The renewable energy systems will be used to replace the LFO and HFO thermal power facilities that are linked to Cameroon's southern interconnected grid. LPSP, LOLP, and TAC are the techno-economic criteria used to evaluate the renewable energy system. Six metaheuristics, MOBO, MSSA, MOALO, MOAVOA, SPEA2, and MOPSO, were employed to ensure the appropriate scaling of these renewable energy systems. The outcomes of the six algorithms were compared. The MOBO algorithm produced the best outcome. Indeed, the TAC achieved with the MOBO algorithm is 0.17%, 0.57%, 0.73%, 7.03%, and 7.26% lower compared to the TACs achieved using the MSSA, MOALO, MOPSO, SPEA2, and MOAVOA algorithms, respectively. In addition, the ideal position of the PV field was investigated. According to the statistics, the locality of Bafoussam had the best total annualized project cost, followed by the city of Yaoundé. The competitiveness of the new renewable energy system was investigated. It demonstrates that the capital necessary to build renewable energy systems may be repaid based on a selling price of 0.12€/kWh for the energy produced by the renewable energy systems.

As renewable energy sources continue to play an increasingly vital role in Cameroon's energy landscape, future research can delve into various avenues to optimize and enhance the integration and utilization of these resources further. One potential direction is the exploration of technological integration and efficiency improvement, where a comprehensive approach is taken to combine multiple renewable energy systems—such as wind, solar, and hydro—in a manner that optimizes their synergies and ensures a stable and sustainable power supply. Advanced control and management systems represent another promising area, with research opportunities lying in the development of systems that can optimize power generation, storage, and distribution in real-time. This would involve considerations such as weather conditions, demand variations, and grid requirements. Additionally, research could delve into hybrid energy systems that combine renewable energy sources with conventional sources like gas or diesel generators, providing a reliable and cost-effective solution for regions with intermittent renewable resources. Another avenue for future research is the exploration of energy storage technologies, including batteries, compressed air energy storage (CAES), and flywheels. These technologies can play a critical role in maintaining grid stability and balancing supply and demand, thus warranting further investigation into their cost-effectiveness and suitability for Cameroon's grid. Lastly, policy and regulatory frameworks can be refined and developed to incentivize investment in renewable energy infrastructure and foster sustainable energy development, while community-level energy solutions, such as microgrids and decentralized energy systems, could be explored to enhance energy access and resilience in rural areas. These future research directions collectively aim to contribute to the sustainable development of Cameroon's energy sector, promoting energy access, mitigating climate change impacts, and fostering a supportive environment for renewable energy adoption.

## Data Availability

The datasets used and/or analysed during the current study available from the corresponding author on reasonable request.

## Appendix

Technical and economic considerations.

**Table:**

PV system	
Model^109^	LONGI LR4-72HPH-450
Rated power	$450 {\text{Wp}}$
NOCT	$45^\circ {\text{C}}$
$\beta$	$0.35\%$
${C}_{cap}^{PV}$^110^	$857{\text{\EUR}}/{\text{kW}}$
${C}_{O\&M}^{PV}$	$1\%$
Life span	$25 {\text{years}}$
PHES system^111^	
${\eta }_{t}\times {\eta }_{p}$	75%
${C}_{cap}^{PHES}$	$5131 {\text{\EUR}}/kW$
Cost of balance	$15 {\text{\EUR}}/kW$
${C}_{cap}^{stor}$	$68 {\text{\EUR}}/kWh$
${C}_{O\&M-f}^{PHES}$	$4.6{\text{\EUR}}/kW$
${C}_{O\&M-V}^{PHES}$	$0.22{\text{\EUR}}{\text{MWh}}$
Ultra-capacitor	
${C}_{cap}^{UC}$^112^	$2000 \$kWh$
${C}_{O\&M}^{UC}$	$1\%$
Life span^112^	Above 25 years
Inverter^113^	
${C}_{cap}^{Inv}$	$210 \$kW$
${C}_{rep}^{Inv}$	$210 \$/kW$
${C}_{O\&M}^{Inv}$	$1\%$
Life span	$15 years$
Economic parameters	
${i}_{n}$	$7\%$
${i}_{f}$	$3\%$
Lifetime of the project	$25 years$
Algorithm parameters	
Iteration	$200$
Population	$200$
